# The cranial and postcranial morphology of *Hutchemys rememdium* and its impact on the phylogenetic relationships of *Plastomenidae* (*Testudinata*, *Trionychidae*)

**DOI:** 10.1186/s13358-024-00315-8

**Published:** 2024-05-24

**Authors:** Léa C. Girard, J. Mark Erickson, Tyler R. Lyson, John W. Hoganson, Walter G. Joyce

**Affiliations:** 1 Department of Geosciences, University of Fribourg, 1700 Fribourg, Switzerland; 2 Department of Geology, St. Lawrence University, Canton, NY 13617 USA; 3 Department of Earth Sciences, Denver Museum of Nature & Science, Denver, CO 80205 USA; 4 North Dakota Geological Survey, Bismarck, ND 58505 USA

**Keywords:** *Trionychia*, *Trionychidae*, *Plastomenidae*, Softshell, Turtle, Taxonomy, Evolution

## Abstract

*Hutchemys rememdium* is a poorly understood softshell turtle (*Trionychidae*) from the mid Paleocene of the Williston Basin of North America previously known only from postcranial remains. A particularly rich collection of previously undescribed material from the Tiffanian 4 North American Land Mammal Age (NALMA) of North Dakota is here presented consisting of numerous shells that document new variation, some non-shell postcrania, and cranial remains, which are described based on 3D models extracted from micro-CT data. Although the observed shell variation weakens previously noted differences with the younger species *Hutchemys arctochelys* from the Clarkforkian NALMA, the two taxa are still recognized as distinct. Parsimony and Bayesian phylogenetic analyses reaffirm the previously challenged placement of *Hutchemys rememdium* within the clade *Plastomenidae*, mostly based on novel observations of cranial characters made possible by the new material and the micro-CT data. The new topology supports the notion that the well-ossified plastron of plastomenids originated twice in parallel near the Cretaceous/Paleogene boundary, once in the *Hutchemys* lineage and once in the *Gilmoremys*/*Plastomenus* lineage. *Hutchemys rememdium* is notable for being the only documented species of trionychid in the mid Paleocene of the Williston Basin. The presence of multiple individuals in a carbonaceous claystone indicates this taxon lived in swamps and lakes and its expanded triturating surface suggests it had a durophagous diet.

**Supplementary Information:**

The online version contains supplementary material available at 10.1186/s13358-024-00315-8.

## Introduction

Softshell turtles (*Pan-Trionychidae*) are an unusual clade of reptiles easily recognized in the fossil record by their reduced carapace that lacks pygals and peripherals, the lack of scutes, a reduced plastron, and a distinctive shell surface texture that allows even shell fragments to be diagnosed to the group (Hutchison et al., 1986; Meylan, 1987; Scheyer et al., 2007; Vitek & Joyce, 2015; Georgalis & Joyce, 2017). The clade has a rich and relatively continuous record in North America since the Santonian (Adrian et al., 2019; Brinkman, 2003; Edgar et al., 2022; Vitek & Joyce, 2015), but apparently originated in Asia during the Early Cretaceous (Georgalis, 2021; Georgalis & Joyce, 2017; Li et al., 2015). The clade has a geographically widespread fossil record with specimens reported from Asia, Europe, and Africa, and to a lesser degree from Australia and South America (Georgalis, 2021; Georgalis & Joyce, 2017; Li et al., 2015; Vitek & Joyce, 2015).

In the Campanian to Eocene record of North America, several pan-trionychid taxa are present that possess expanded carapacial callosities that cover much of the ribs and plastra. In some taxa, the hyo-, hypo-, and xiphiplastral callosities are expanded such that they fully suture with one another to form a solid shield similar to the plastron of other turtles. Hay (1902, 1908) united all turtles with expanded plastra into *Plastomenidae*, but there has been little agreement over the years as to which taxa belong to this group (see Vitek & Joyce, 2015 for summary). A number of fossils from the Paleogene of Asia have historically been referred to *Plastomenidae*, but have since been shown to have no apparent relationship (Danilov et al., 2017; Georgalis & Joyce, 2017). The name is now defined as referring to the most inclusive clade of turtles that includes the Eocene *Plastomenus thomasii*, but no species of extant turtle (Joyce et al., 2021).

The Maastrichtian softshell turtle *Gilmoremys lancensis* (Gilmore, 1916) was originally described as a trionychine but the discovery of cranial material has since shown that this taxon is closely related to *Plastomenus thomasii*, as both taxa have an elongate skull with a secondary palate, a spatulate mandible, and reduced to absent postorbitals (Evers et al., 2023; Joyce & Lyson, 2011; Joyce et al., 2016). The two taxa also share the presence of a striated carapace and two hyoplastral processes (Joyce & Lyson, 2011; Joyce et al., 2016). Together with other named species of *Gilmoremys* and *Plastomenus* (Joyce et al., 2018; Lyson et al., 2021), this implies the gradual acquisition of the plastomenid body plan from the Campanian to the Eocene.

Several trionychids coeval with the *Gilmoremys*/*Plastomenus* lineages were recently united under the term *Hutchemys* based on their unique plastral morphology (Joyce & Lyson, 2011; Joyce et al., 2009). Like *Plastomenus* spp., these taxa have a fully formed plastron that lacks fontanelles, but in addition the entoplastron forms a large, rectangular callosity that is sutured to the expanded hyoplastra. Intriguingly, this taxon possesses only a single hyoplastral process, a derived trait that also occurs in a number of coeval taxa, such as the Campanian *Aspideretoides foveatus* or the Paleocene *Atoposemys superstes* (Gardner et al., 1995; Hutchison, 2013). This implies the acquisition of a well-ossified shell independent from that of *Plastomenus thomasii*. Some current phylogenies suggest that both lineages combined form the clade *Plastomenidae* (e.g., Joyce et al., 2018; Lyson et al., 2021; but see Evers et al., 2023 suggesting paraphyly), but cranial characters are absent that would support this arrangement, as the skull of no taxon with a single hyoplastral process is described sufficiently.

We here describe a rich collection of new fossil turtle material referrable to *Hutchemys rememdium* (Hutchison, 2009; Joyce et al., 2009), a species primarily named based on a partial skeleton from the Torrejonian of Montana. In addition to firmly establishing the presence of this taxon in the Tiffanian of the Williston Basin, the new material is significant by including numerous cranial remains that we describe based on 3D models obtained from µCT scans. Although the cranial morphology of *Hutchemys rememdium* is superficially different from the *Gilmoremys*/*Plastomenus* lineage, our phylogenetic analysis overall corroborates recent hypotheses regarding the composition of *Plastomenidae*.

## Geological settings

**Fig. 1:**
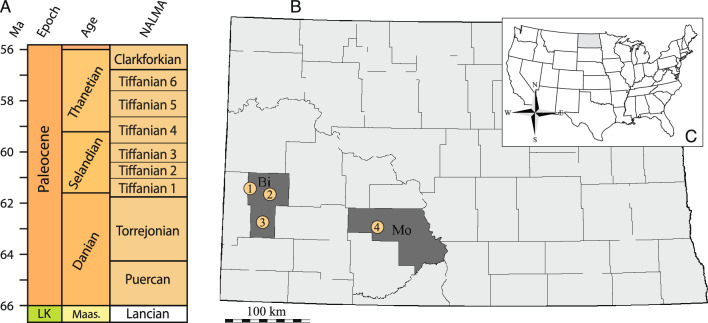
**A**, Graphic of the North American Land Mammal Ages (NALMA) correlated to Chronostratigraphic stage (Based on Speijer et al., 2020) **B,** A map of North Dakota highlighting the counties that yielded the material described herein **C**, A map of the United States with North Dakota in gray. **1**, Wannagan Creek Locality; **2**, Ash Coulee Quarry; **3**, Tracey Mountain Locality; **4**, Judson Locality. *Bi* Billings County, *LK* Late Cretaceous, *Maas* Maastrichtian, *Ma* millions of years ago, *Mo* Morton County

This study is based on a large sample of *Hutchemys rememdium* that had been collected by the North Dakota Geological Survey (NDGS) and Science Museum of Minnesota (SMM) over the course of the last several decades in the western half of North Dakota, U.S.A. (Fig. 1B). All specimens originate from the Sentinel Butte and Bullion Creek formations of the Fort Union Group. Although some ambiguity remains, all localities are correlated with the Tiffanian 4 North American Land Mammals Age (NALMA; Fig. 1A; see below), which approximates to the Middle to Late Paleocene boundary. As we ultimately conclude that all specimens represent a single taxon, we describe all fossils in concert, but mostly figure them by locality. A brief summary of the geological setting of all localities is provided below. More detailed information can be obtained from the relevant institutions.

*Ash Coulee Quarry (NDGS)* The Ash Coulee Quarry is located in badlands exposed near the town of Fairfield in Billings County, North Dakota (Fig. 1). The turtles from the Ash Coulee Quarry were extracted from beds of poorly indurated dark grey shales, in particular a 10–20 cm thick layer of carbonaceous claystone called the “Turtle Bed.” Sediments are indicative of a deltaic swamp and lake environment (Hoganson, 1997; Kays, 1999; Kays et al., 1996). The Ash Coulee Quarry is located within the lower third of the Sentinel Butte Formation (Kays, 1999; Kays et al., 1996, 1998). No mammals have been recovered that would allow the quarry to be confidently assigned to a particular NALMA. Indeed, although mammals have been recovered from the entire formation, only a few have been evaluated formally and the formation itself remains poorly studied in general. However, preliminary estimates suggest a Tiffanian 4 NALMA for the lower third of the Sentinel Butte Formation (Kihm & Hartman, 2004). The quarry produced hundreds of remains of turtles, mostly *H*. *rememdium*, but also rare remnants of the chelydrid *Protochelydra zangerli* (Kays, 1999). The turtle fauna was initially attributed to *Plastomenus* (Kays et al., 1996)*,* but later to *Plastomenidae* indet. (Kays, 1999). We here restrict ourselves to describing specimens with the greatest anatomical information, including two crania, four mandibles, numerous shells, and select limb bones.

*Tracy Mountain (NDGS)* The Sentinel Butte Formation is exposed at the Tracy Mountain Site, which is located near the town of Fryburg in Billings County, North Dakota (Fig. 1). Remains of vertebrates, invertebrates, and plants originate from an organic-rich carbonaceous claystone deposit, interpreted to represent a swamp environment. This site yielded near-complete skeletons of champsosaurs and is therefore also known as the “Champsosaur Fossil Site” (Hoganson, 1997; Hoganson & Campbell, 1995). No mammals or other turtles have been found at the locality. However, the high concentration of nearby fossil localities in the lower third of the Sentinel Butte Formation (Kihm & Hartman, 2004) makes a Tiffanian 4 NALMA, or slightly younger, highly plausible.

*Wannagan Creek (SMM)* Wannagan Creek is located in the upper breaks (i.e., badlands) of the Missouri River near Theodore Roosevelt National Park, Billings County, North Dakota (Erickson, 1982, 1999; Fig. 1). The quarry is stratigraphically located in the Bullion Creek Formation, about 20 m below its upper contact with the Sentinel Butte Formation. The Wannagan Creek locality is a particularly rich Lagerstätte that was systematically quarried over the course of three decades (Erickson, 1999). The fossil assemblage includes a variety of freshwater taxa, including turtles, crocodilians, champsosaurs, snakes, lizards, amphibians, and fish, as well as mammals, birds, and a wide variety of plants (Erickson, 1999). Although the rich mammalian fauna should allow placing this quarry within a specific NALMA with confidence, a formal evaluation is still outstanding. However, a preliminary assessment suggests a Tiffanian 4 NALMA (Erickson, 1999). The Bullion Creek Formation at Wannagan consists of clay and silty marls below lignite beds interbedded with plant remains and mudstones indicative of a depositional environment of swamps and lakes (Erickson, 1991, 1999; Wallick, 1984). Erickson (2012) reported the presence of the chelydrid *Protochelydra zangerli*, two trionychids, a “polycryptodire,” and an emydid. After having viewed all material as part of this study, we confirm the presence of *Protochelydra zangerli*, but otherwise find only a single trionychid, the rare shell material of *Hutchemys rememdium* reported herein, an indeterminate macrobaenid, and the potential chelydroid *Cardichelyon rogerwoodi* (Hutchison, 2013; Joyce & Claude, 2020), respectively.

While quarrying at Wannagan, SMM field crews prospected nearby outcrops of the Bullion Creek and Sentinel Butte formations, resulting in the recovery of additional remains of *H*. *rememdium*, the best preserved of which are described herein. Although stratigraphic data is currently missing beyond indications of the formation, we presume these specimens to also be from the Tiffanian 4 NALMA.

*Judson Locality (SMM)* The Judson Locality consists of four distinct sites that are located in Morton County, North Dakota (Fig. 1). The locality is located 24 m above the base of the Bullion Creek Formation (Holtzman, 1978; Kihm & Hartman, 2004). The fossil mammals found at this site are indicative of a Tiffanian 4 NALMA (Kihm & Hartman, 2004). The sediments consist of unconsolidated, fine-grained sands with varying amounts of clay. The locality yielded no other turtles.

## Materials and methods

### Digital anatomy

Two crania (NDGS 10019 and NDGS 10029), four mandibles (NDGS 10034, NDGS 10084, NDGS 10329, NDGS 11788), and various postcranial elements were scanned at the headquarters of North Star Imaging in Rogers, Minnesota, using a X3000 and a CXMM50 micro-CT (μCT) scanners. The resulting 8-bit tiff stacks were processed with the software Materialise Mimics v.24. Bone by bone semi-automatic segmentation was performed to obtain individual ply models of each bone and major canals with osteological preservation. Shells, pectoral and pelvic girdles were scanned using a portable surface scanner Artec Space Spider at NDGS and SMM. The scans were acquired and treated with the software Artec Studio 16 Professional. All models with scanning parameters are available at MorphoSource (https://www.morphosource.org/projects/000594396), and a table is provided in Additional file 1: Material S1 with individual links to each 3D model.

### Comparative anatomy

We mostly compare the skull anatomy of *Hutchemys rememdium* to that of other well-described plastomenids, in particular the Late Cretaceous (Maastrichtian) *Gilmoremys lancensis* (as described by Joyce & Lyson, 2011; Joyce et al., 2016) and the Eocene *Plastomenus thomasii* (as described Evers et al., 2023). The skull descriptions of *G*. *lancensis* are based on external observations of multiple specimens while the description of *P*. *thomasii* is based on a µCT scan of a single skull. We additionally compare *Hutchemys rememdium* to extant species of trionychids by reference to Meylan (1987) and µCT based 3D models available to us of *Amyda cartilaginea* (FMNH 244117), *Apalone ferox* (MVZ 241534), *Apalone spinifera* (FMNH 22178), *Chitra chitra* (NHMUK 65.12.16.1), *Cycloderma frenatum* (NHMUK 84.2.4.1), *Cyclanorbis senegalensis* (NHMUK 65.5.9.21), *Dogania subplana* (PCHP 2919), *Lissemys ceylonensis* (NMB 2398), *Lissemys punctata* (SMF 74141), *Nilssonia formosa* (NHMW 38631), *Palea steindachneri* (MTD 33802), *Pelochelys cantorii* (NMB 11985, PCHP 4974)*, Pelodiscus sinensis* (NMB 1438), *Rafetus euphraticus* (NHMW 132), and *Trionyx triunguis* (PCHP 4559). All 3D models will be made public in the near future, but can be requested immediately. Unless specified otherwise, our cranial nomenclature follows that of Gaffney (1972).

### Phylogenetic analyses

To investigate the phylogenetic relationships of *Hutchemys rememdium*, we employ the trionychid matrix of Evers et al. (2023), which consists of 40 taxa and 95 characters, with fossil taxa mostly consisting of stem trionychids and plastomenids, as well as the North American taxon *Axestemys infernalis* whose phylogenetic position remains unresolved. No taxa or characters were added to the matrix, but several scorings for *Hutchemys rememdium* were updated using the material described herein. The adjustments can be summarized as the following: ch. 15: 2 → 1, ch. 21: 1 → 2, ch. 23: ? → 2, ch. 28: ? → 1, ch. 29: ? → 1, ch. 30: ? → 1, ch. 31: ? → 2, ch. 32: ? → 1, ch. 34: ? → 2, ch. 36: ? → 1, ch. 37: ? → 2, ch. 38: ? → 2, ch. 39: ? → 0, ch. 40: ? → 3, ch. 41: ? → 1, ch. 42: ? → 1, ch. 45: ? → 1, ch. 46: ? → 1, ch. 47: ? → 1, ch. 48: ? → 1, ch. 49: ? → 1, ch. 50: ? → 1, ch. 51: ? → 1, ch. 53: ? → 1, ch. 54: ? → 1, ch. 55: ? → 1, ch. 80: ? → 1, ch. 81: ? → 0, ch. 82: ? → 0&1, ch. 83: ? → 0, ch. 94: ? → 0 ch. 95: ? → 1, ch. 96: ? → 1, ch. 97: ? → 1, ch. 99: ? → 1, ch. 101: ? → 0, ch. 102: ? → 1, ch. 103: ? → 0, ch. 104: ? → 1, ch. 105 ? → 1, ch. 106: ? → 0, ch. 108: ? → 1, ch. 109: ? → 2, ch. 116: ? → 1. We furthermore implemented the following changes for other taxa: ch. 15: 2 → 1 for *Hutchemys* spp. and *Helopanoplia distincta*, ch. 21: 1 → ? For *H*. *tetanetron* and *Aspideretoides foveatus*, and ch. 80: 0 → 1 for *T*. *triunguis*. In order to explore the impact of our modified scoring upon the phylogenetic placement of *Hutchemys rememdium*, we otherwise closely replicated the parsimony and Bayesian analyses of Evers et al. (2023). The updated matrix and list of characters are available in the  Additional file 2.

*Bayesian analyses* Bayesian tip-dating analysis was performed using MrBayes 3.2.7 (Ronquist et al., 2009) following the settings used by Evers et al. (2023). Characters 1, 3, 5, 16, 18–20, 22, 31, 40, 44, 53–54, 59, 77, 102, 108–110 were treated as ordered morphoclines. *Adocus lineolatus* was set as the outgroup. *Nemegtemys conflata*, formerly identified as a rogue taxon (Evers et al., 2023), was pruned from the analysis. Extant trionychid relationships were constrained using the following molecular topology recovered by Thomson et al. (2021): (*Adocus_lineolatus* (*Carettochelys_insculpta*(((*Cyclanorbis_elegans Cyclanorbis_senegalensis*)(*Lissemys_punctata* (*Cycloderma_aubryi Cycloderma_frenatum*)))((*Trionyx_triunguis*(*Chitra_indica Pelochelys_bibroni*))((*Rafetus_euphraticus* (*Apalone_mutica* (*Apalone_ferox Apalone_spinifera*)))(*Pelodiscus_sinensis*(*Palea_steindachneri* (*Dogania_subplana* (*Amyda_cartilaginea* (*Nilssonia_gangetica* (*Nilssonia_formosa Nilssonia_hurum*))))))))))). In addition, *Adocus lineolatus* and *Carettochelys insculpta* were constrained to be recovered outside of *Pan-Trionychidae*. Mkv + G with a relaxed clock was used as the sampling model. This tree model is a fossilized-birth–death with default settings. The analysis was performed using a MCMCMC algorithm, two independent runs of four chains with a temperature of 0.05, and a number of branch swapping of 3. The analysis produced 50,000,000 generations with sampling every 500 generations and a burn in of 25%.

*Parsimony analyses* Parsimony analyses were performed using TNT 1.5 (Goloboff & Catalano, 2016). Following the methods used by Evers et al., (2023; Additional file 3), the analyses were conducted using New Technology Search with the initial level set to 30 and the minimum length hit 30 times and all search algorithms enabled. The memory was manually increased to 99,999 trees. As with the Bayesian analysis, all morphoclines were ordered, a molecular backbone (i.e., constraint) was implemented following Thomson et al. (2021), and *Adocus lineolatus* was set as the outgroup. Unweighted analysis and weighted K = 12 analyses were performed. To improve the tree resolution, *Nemegtemys conflata*, previously identified as a rogue taxon, was pruned from the analysis.

## Systematic paleontology

*Testudinata* Klein, 1760 (sensu Joyce et al., 2020).

*Pan-Trionychidae* Joyce et al., 2004 (sensu Joyce et al., 2021).

*Plastomenidae* Hay, 1902 (sensu Joyce et al., 2021).

*Hutchemys* Joyce et al., 2009

**Type species:** *Hutchemys rememdium* Joyce et al., 2009.

**Revised diagnosis:** *Hutchemys* can be identified as a member of *Trionychidae* by the following characters: absence of peripherals, pygals, suprapygals and shell scutes, presence of sculpturing on the metaplastic portions of the shell, and a boomerang shaped entoplastron. *Hutchemys* is differentiated from other trionychids, including “*Hutchemys*” *walkerorum*, by the presence of an octagonal neural 2 and an enlarged, rectangular entoplastral callosity that is laterally and posteriorly sutured to the hyoplastra.

*Hutchemys rememdium* Joyce et al., 2009

(Figs. 2, 3, 4, 5, 6, 7, 8, 9, 10, 11, 12, 13, 14, 15, 16).

**Fig. 2:**
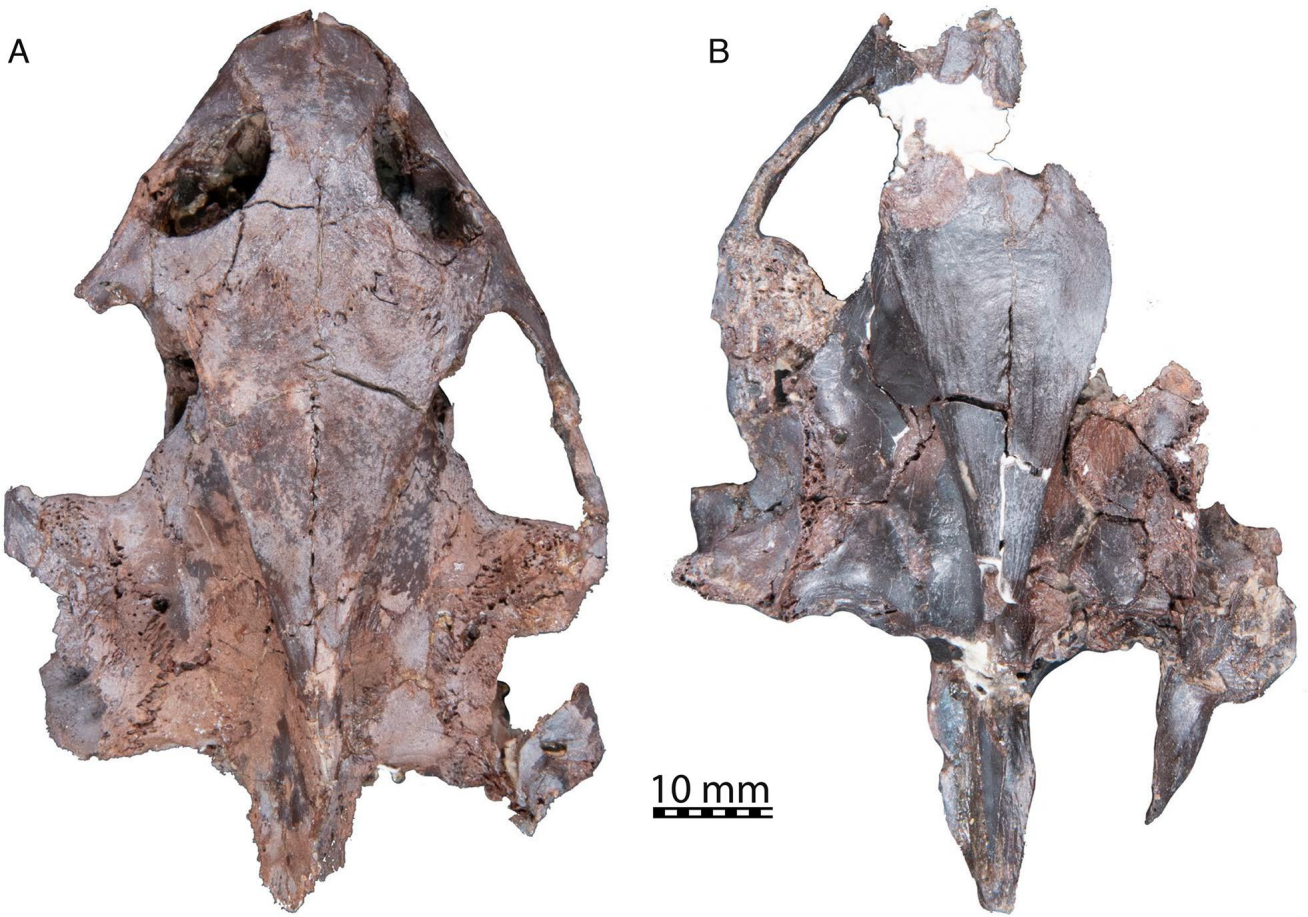
Photographs of *Hutchemys rememdium* crania in dorsal view, Ash Coulee Quarry, Sentinel Butte Formation, Tiffanian 4, Billings County, North Dakota. **A**, NDGS 10019; **B**, NDGS 10029

**Fig. 3:**
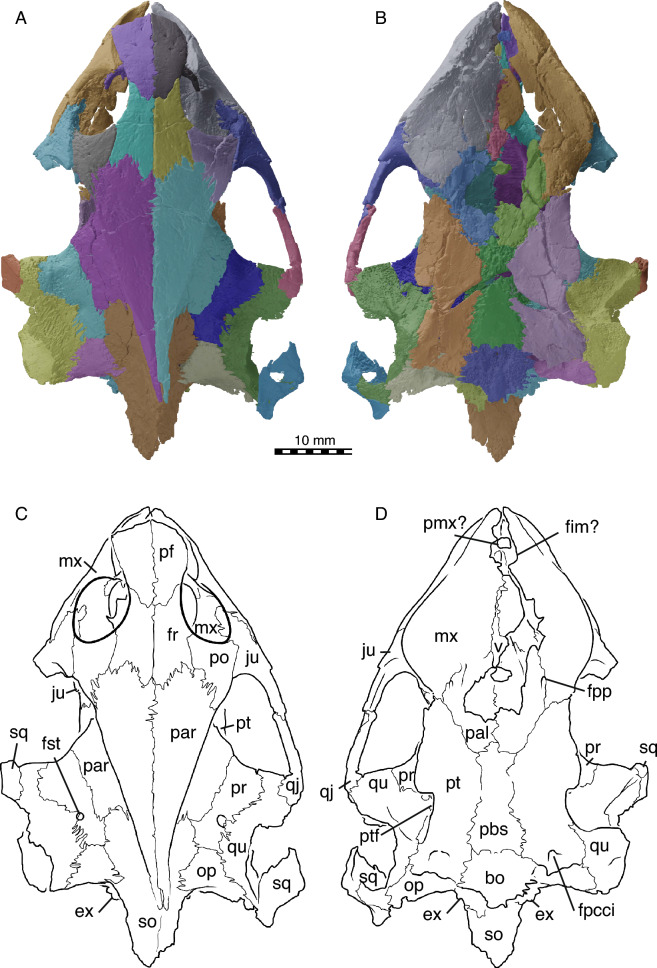
NDGS 10019, cranium of *Hutchemys rememdium*, Ash Coulee Quarry, Sentinel Butte Formation, Tiffanian 4, Billings County, North Dakota. **A**, 3D model in dorsal view; **B**, 3D model in ventral view; **C**, line drawing in dorsal view; **D**, line drawing in ventral view. *bo* basioccipital, *ex* exoccipital, *fim* intermaxillary foramen, *fpcci* foramen posterius canalis carotici interni, *fpp* foramen palatinus posterius, *fr* frontal, *fst* foramen stapedio-temporale, *ju* jugal, *mx* maxilla, *op* opisthotic; *pal* palatine, *par* parietal, *pf* prefrontal, *pmx* premaxilla, *po* postorbital, *pr* prootic, *pt* pterygoid, *ptf* pterygoid flange, *qj* quadratojugal, *qu* quadrate, *so* supraoccipital, *sq* squamosal, *v* vomer

**Fig. 4:**
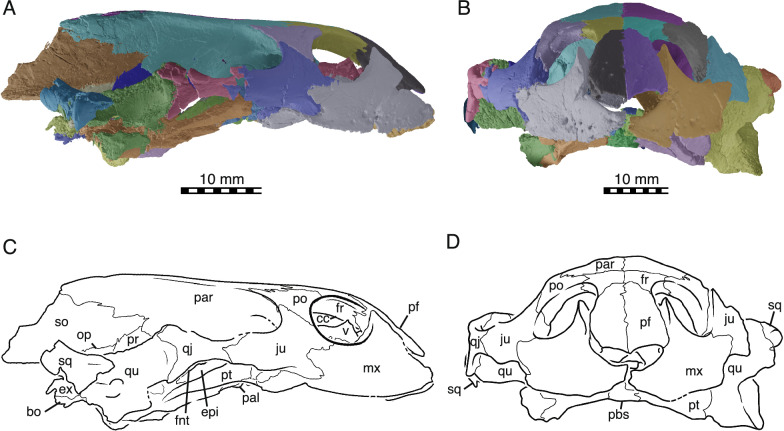
NDGS 10019, cranium of *Hutchemys rememdium*, Ash Coulee Quarry, Sentinel Butte Formation, Tiffanian 4, Billings County, North Dakota. **A**, 3D model in right lateral view. **B**, 3D model in anterior view. **C**, line drawing in right lateral view. **D**, line drawing in anterior view. *bo* basioccipital, *cc* crista cranii, *epi* epipterygoid, *ex* exoccipital, *fnt* foramen nervi trigemini, *fr* frontal, *ju* jugal, *mx* maxilla, *op* opisthotic, *pal* palatine, *par* parietal, *pbs* parabasisphenoid, *pf* prefrontal, *po* postorbital, *pr* prootic, *pt* pterygoid, *qj* quadratojugal, *qu* quadrate, *so* supraoccipital, *sq* squamosal, *v* vomer

**Fig. 5:**
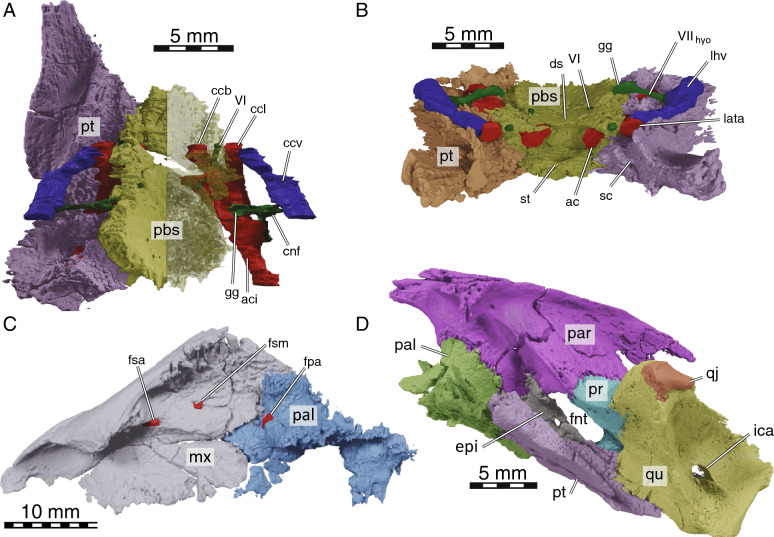
Details on the circulatory and innervation structures in NDGS 10019, cranium of *Hutchemys rememdium*. 3D model of the partial basicranium and reconstructed arteries and nerves in **A**, dorsal and **B**, frontal views. **C**, 3D model of the right maxilla and reconstructed arteries in mediodorsal view. **D**, 3D model of the left trigeminal foramen and adjacent bones in lateral view. *ac* arteria caroticus cerebralis, *aci* arteria carotis interna, *bs* parabasisphenoid, *ccb* canalis caroticus basisphenoidalis, *ccl* canalis caroticus lateralis, *ccv* canalis cavernosus, *cnf* canalis nervus facialis, *ds*, dorsum sellae, *epi* epipterygoid, *fsa* foramen supraalveolar, *fnt* foramen nervi trigemini, *fsm* foramen supramaxillare, *gg* geniculate ganglion, *ica* incisura columnella auris, *lata* lateral artery, *lhv* lateral head vein, *mx* maxilla, *pal* palatine, *par* parietal, *pr* prootic, *pt* pterygoid, *qj* quadratojugal, *qu* quadrate, *sc* sulcus cavernosus, *st* sella turcica, *VI* abducen nerve, *VII*_*hyo*_ hyomandibular branch of the facial nerve

**Fig. 6.**
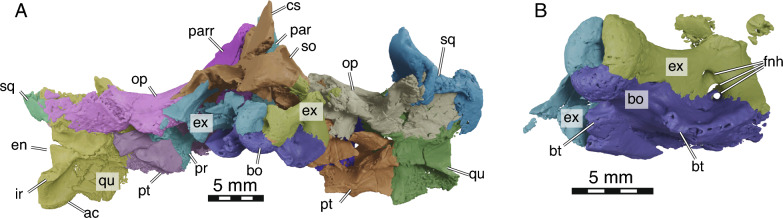
3D models of NDGS 10029, partial cranium of *Hutchemys rememdium*, Ash Coulee Quarry, Sentinel Butte Formation, Tiffanian 4, Billings County, North Dakota. **A**, posterior view; **B**, occipital condyle in detail in ventroposterolateral view. *ac* articular condyle, *bo* basioccipital, *bt* basioccipital tubercule, *cs* crista supraoccipitalis, *en* Eustachian notch, *ex* exoccipital, *fnh* foramina nervi hypoglossi, *ir* infolding ridge, *op* opisthotic, *par* parietal, *pr* prootic, *pt* pterygoid, *qu* quadrate, *so* supraoccipital, *sq* squamosal

**Fig. 7:**
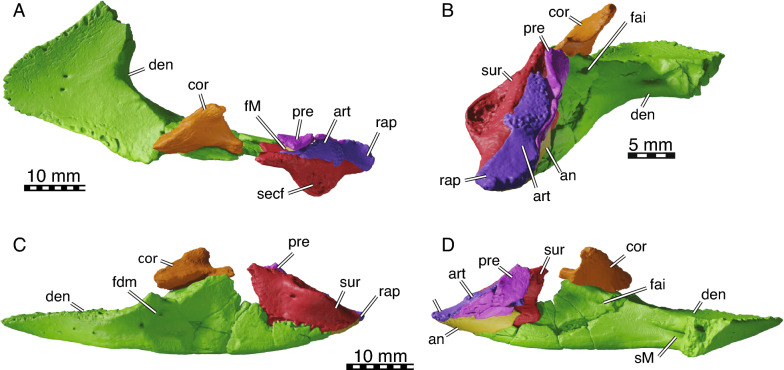
3D models of NDGS 10034, partial mandible of *Hutchemys rememdium*, Ash Coulee Quarry, Sentinel Butte Formation, Tiffanian 4, Billings County, North Dakota. **A**, dorsal view; **B**, posterior view; **C**, left lateral view; **D**, right medial view. *an*, angular, *art* articular, *cor* coronoid, *den* dentary, *fai* foramen alveolare inferius, *fdm* foramen dentofaciale majus, *fM* fossa Meckelii, *pre* prearticular, *rap* retroarticular process, *secf* surangular ectocondylar flange, *sM* sulcus Meckelii, *sur* surangular

**Fig. 8:**
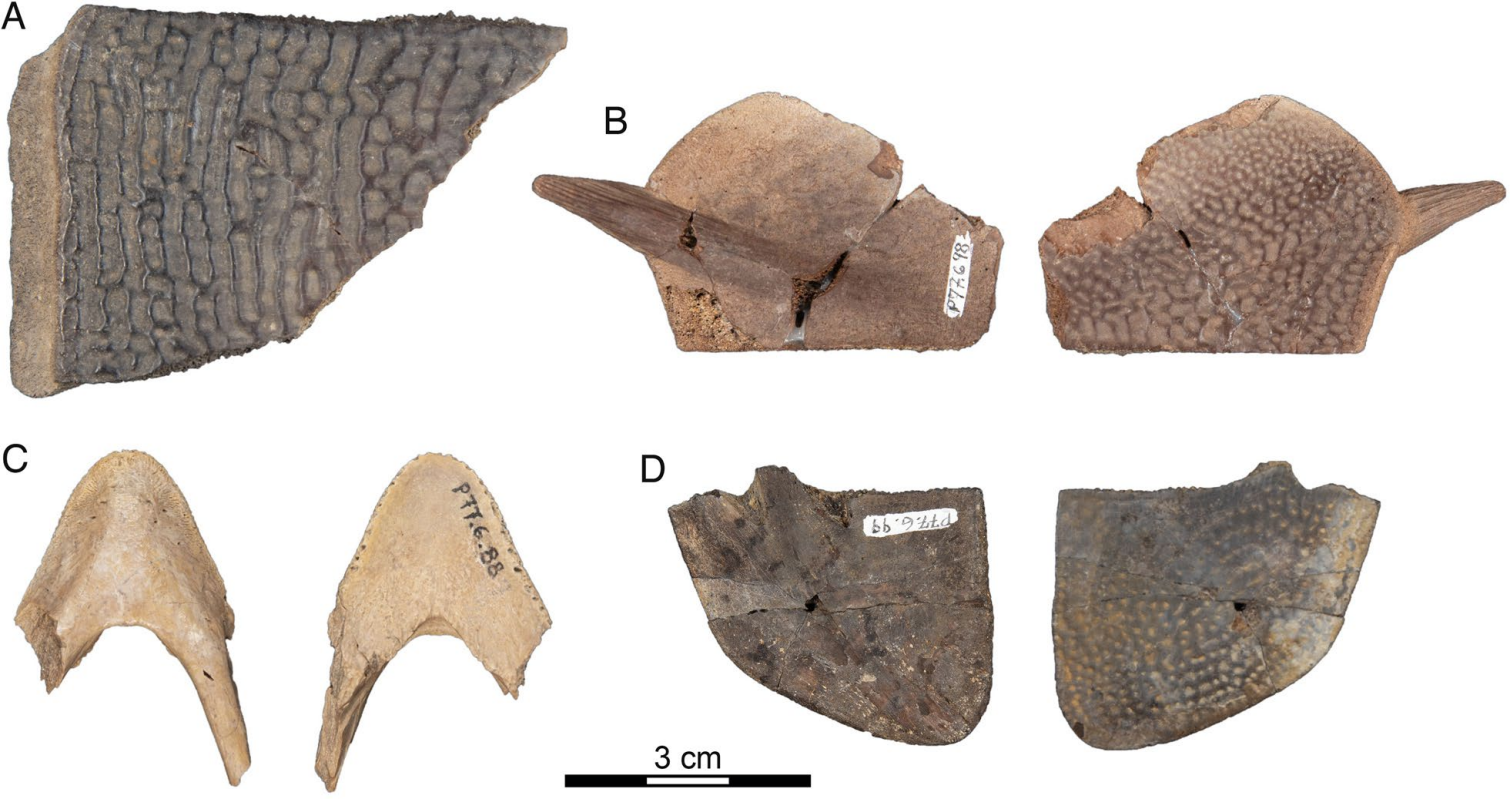
*Hutchemys rememdium*, Judson Locality, Bullion Creek Formation, Tiffanian 4, Morton County, North Dakota. **A**, SMM P77.6.100, costal fragment; **B**, SMM P77.6.98, a partial left hyoplastron; **C**, SMM P77.6.88, partial dentary; **D**, SMM P77.6.99, left xiphiplastron

**Fig. 9:**
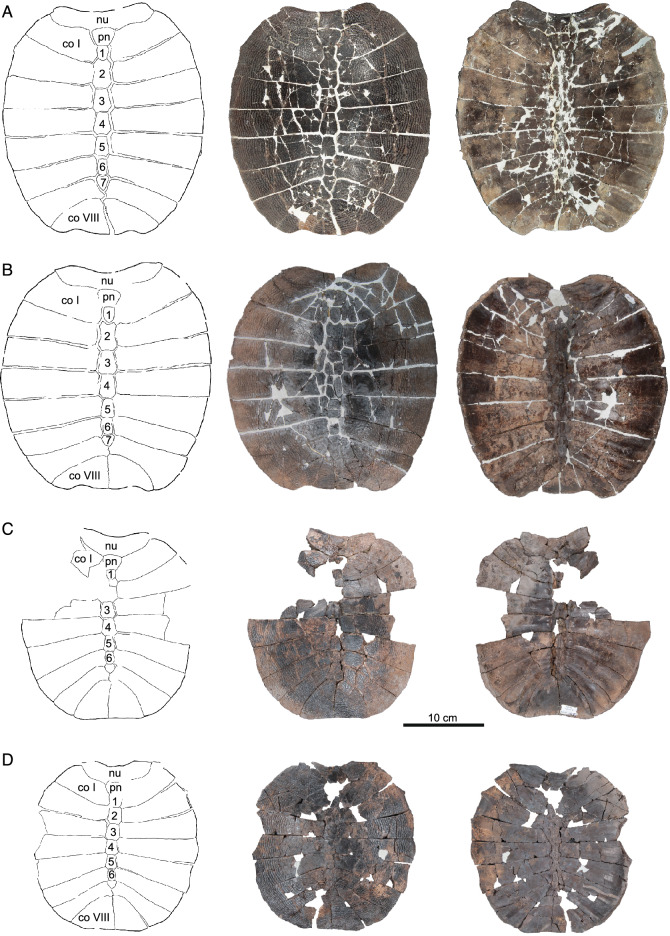
*Hutchemys rememdium*, Ash Coulee Quarry, Sentinel Butte Formation, Tiffanian 4, Billings County, North Dakota. **A**, NDGS 1203, line drawing (left) and photographs of carapace in dorsal (middle) and ventral (right) views; **B**, NDGS 10028, line drawing (left) and photographs of carapace in dorsal (middle) and ventral (right) views; **C**, NDGS 10625, line drawing (left) and photographs of partial carapace in dorsal (middle) and ventral (right) views; **D**, NDGS 11879, line drawing (left) and photographs of partial carapace in dorsal (middle) and ventral (right) views. *co* costal, *nu* nuchal, *pn* preneural. Arabic numerals denote neurals

**Fig. 10:**
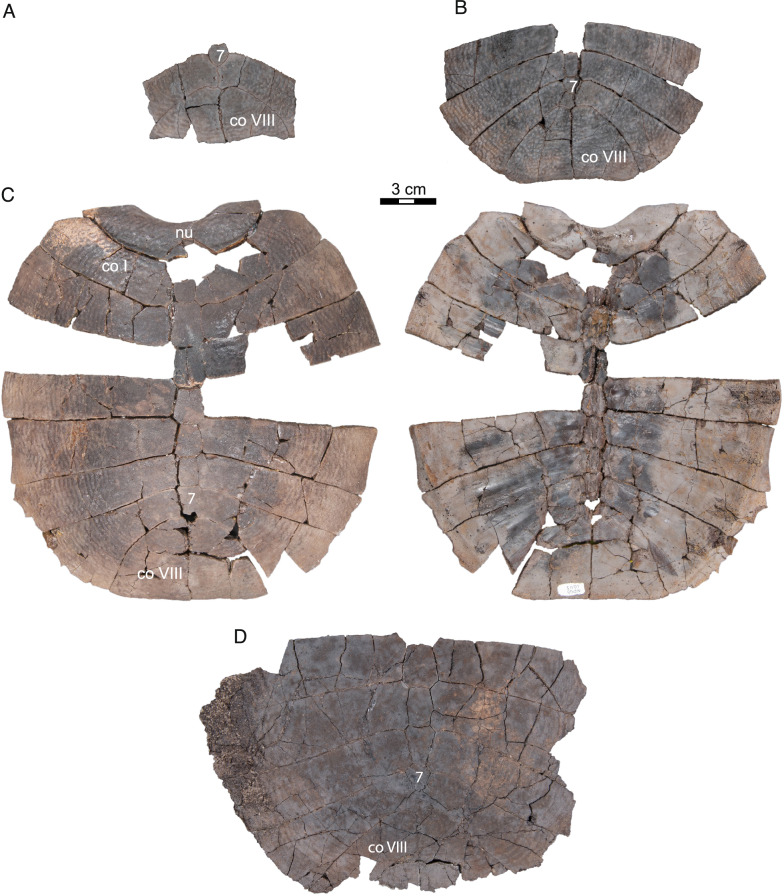
*Hutchemys rememdium*, Ash Coulee Quarry, Sentinel Butte Formation, Tiffanian 4, Billings County, North Dakota. **A,** NDGS 12215 a partial carapace in dorsal view; **B**, NDGS 10057 a partial carapace in dorsal view; **C**, NDGS 10115 an incomplete carapace in dorsal (left) and ventral (right) views; **D**, NDGS 11790 a partial carapace in dorsal view. *co* costal, *nu* nuchal. Arabic numerals denote neurals. Roman numerals denote costals

**Fig. 11:**
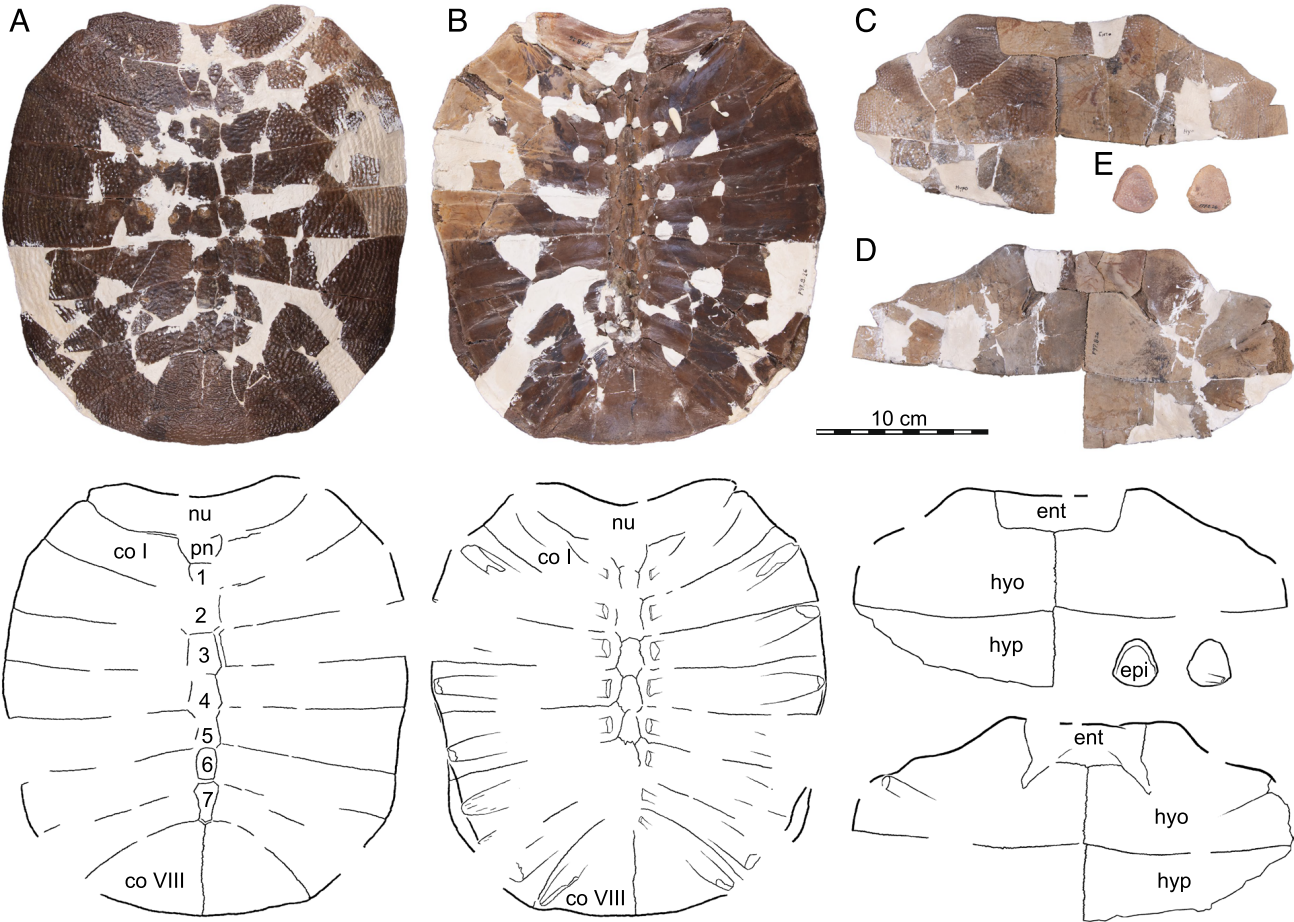
*Hutchemys rememdium*, SMM P97.8.26, near Wannagan Creek, Sentinel Butte Formation, Tiffanian 4, Billings County, North Dakota. Shell and partial plastron SMM P97.8.26. **A**, photograph (top) and line drawing (bottom) of carapace in dorsal view; **B**, photograph (top) and line drawing (bottom) of carapace in ventral view; **C**, photograph and line drawing of plastron in ventral view; **D**, photograph and line drawing of plastron in dorsal view; **E**, photograph and line drawing of epiplastra in ventral (left) and dorsal (right) view. *co* costal, *ent* entoplastron, *epi* epiplastron, *hyo* hyoplastron, *hyp* hypoplastron, *nu* nuchal, *pn* preneural. Arabic numerals denote neurals. Roman numerals denote costals

**Fig. 12:**
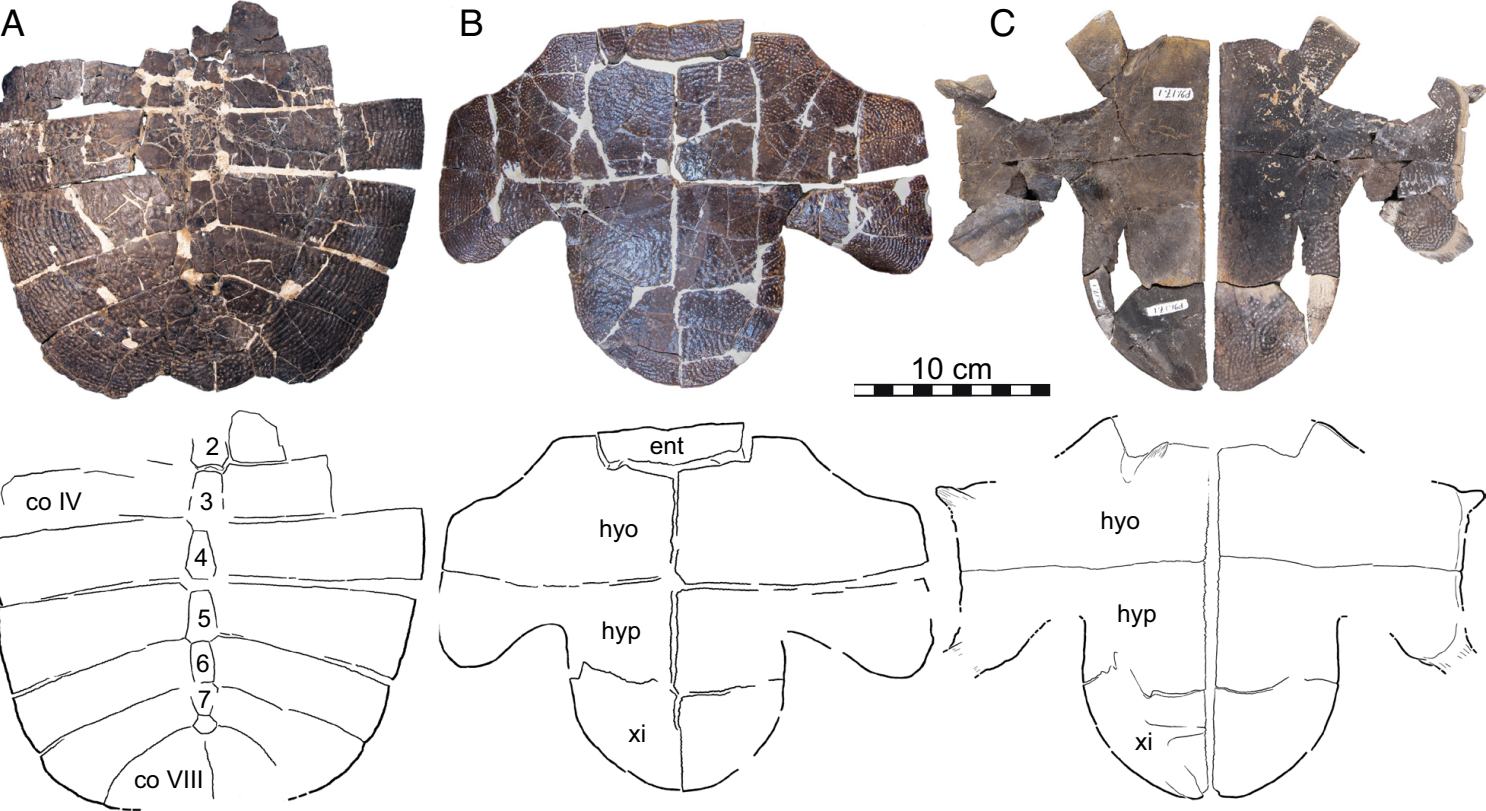
*Hutchemys rememdium* from near Wannagan Creek, Bullion Creek Formation, Tiffanian 4, Billings County, North Dakota. **A**, SMM P91.17.1.1, photographs (top) and line drawings (bottom) of carapace in dorsal view; **B**, SMM P91.17.1.2, photographs (top) and line drawings (bottom) of a plastron lacking epiplastra in ventral view; **C**, SMM P91.17.1.3, photographs (top) and line drawings (bottom) of a partial plastron in dorsal (left) and ventral (right) views. *co* costal, *ent* entoplastron, *hyo* hyoplastron, *hyp* hypoplastron, *xi* xiphiplastron. Numbers denote neurals

**Fig. 13.**
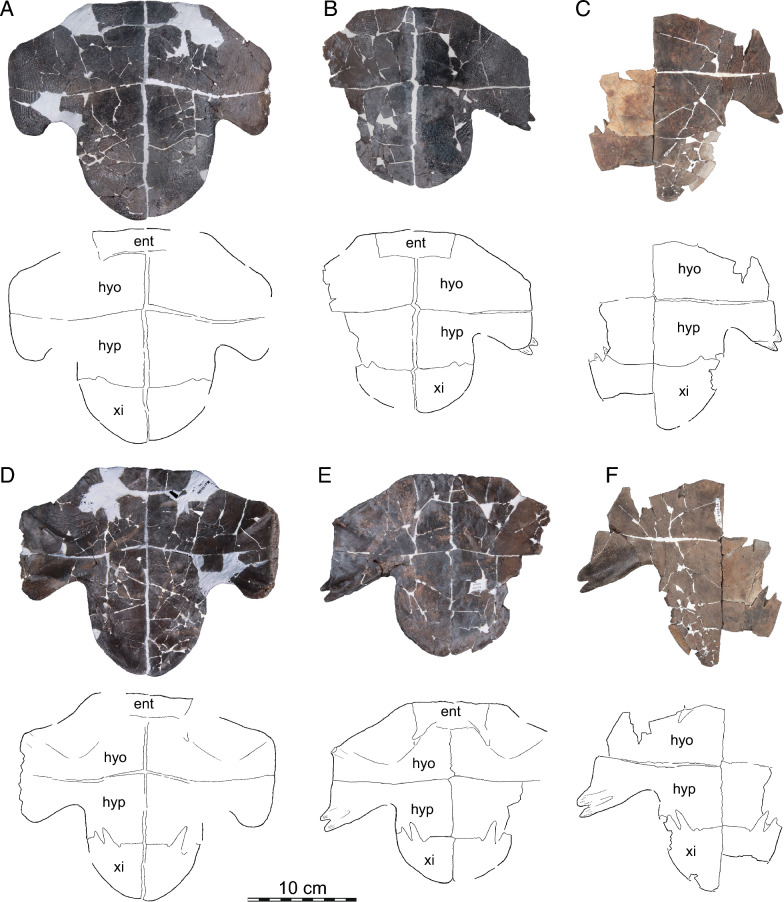
*Hutchemys rememdium*, Ash Coulee Quarry, Sentinel Butte Formation, Tiffanian 4, Billings County, North Dakota. Photographs (top) and line drawings (bottom) of ento-, hyo-, hypo-, and xiphiplastra: **A**, NDGS 1758 in ventral view; **B**, NDGS 10023 in ventral view; **C**, NDGS 18196 in ventral view; **D**, NDGS 1758 in dorsal view; **E**, NDGS 10023 in dorsal view, **F**, NDGS 18196 in dorsal view. *ent* entoplastron, *hyo* hyoplastron, *hyp* hypoplastron, *xi* xiphiplastron

**Fig. 14:**
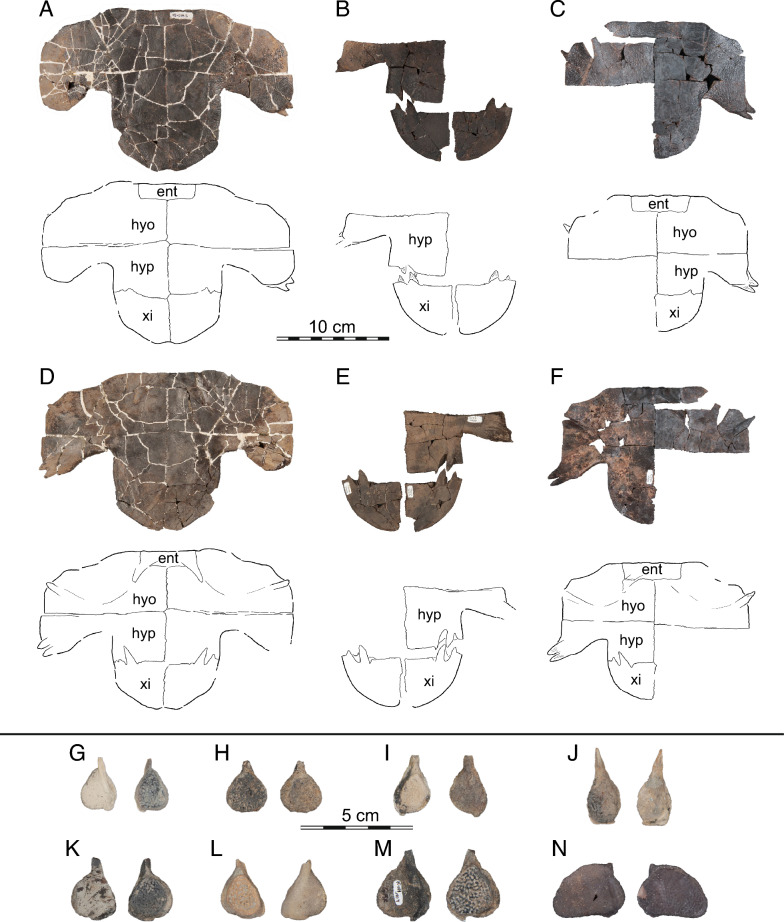
*Hutchemys rememdium*, Ash Coulee Quarry, Sentinel Butte Formation, Tiffanian 4, Billings County, North Dakota. Photographs (top) and line drawings (bottom) of ento-, hyo-, hypo-, and xiphiplastra in ventral (left) and dorsal (right) views: **A**, NDGS 10001 in ventral view; **B**, NDGS 10056 in ventral view; **C**, NDGS 10071 in ventral view; **D**, NDGS 10001 in dorsal view; **E**, NDGS 10056 in dorsal view; **F**, NDGS 10071 in dorsal view. Photographs of epiplastra in dorsal (smooth) and ventral (textured) view: **G**, NDGS 9947; **H**, NDGS 11689; **I**, NDGS 9989; **J**, NDGS 11681; **K** NDGS 11691; **L** NDGS 9975, **M** NDGS 11685; **N** NDGS 12226. *ent* entoplastron, *hyo* hyoplastron, *hyp* hypoplastron, *xi* xiphiplastron

**Fig. 15:**
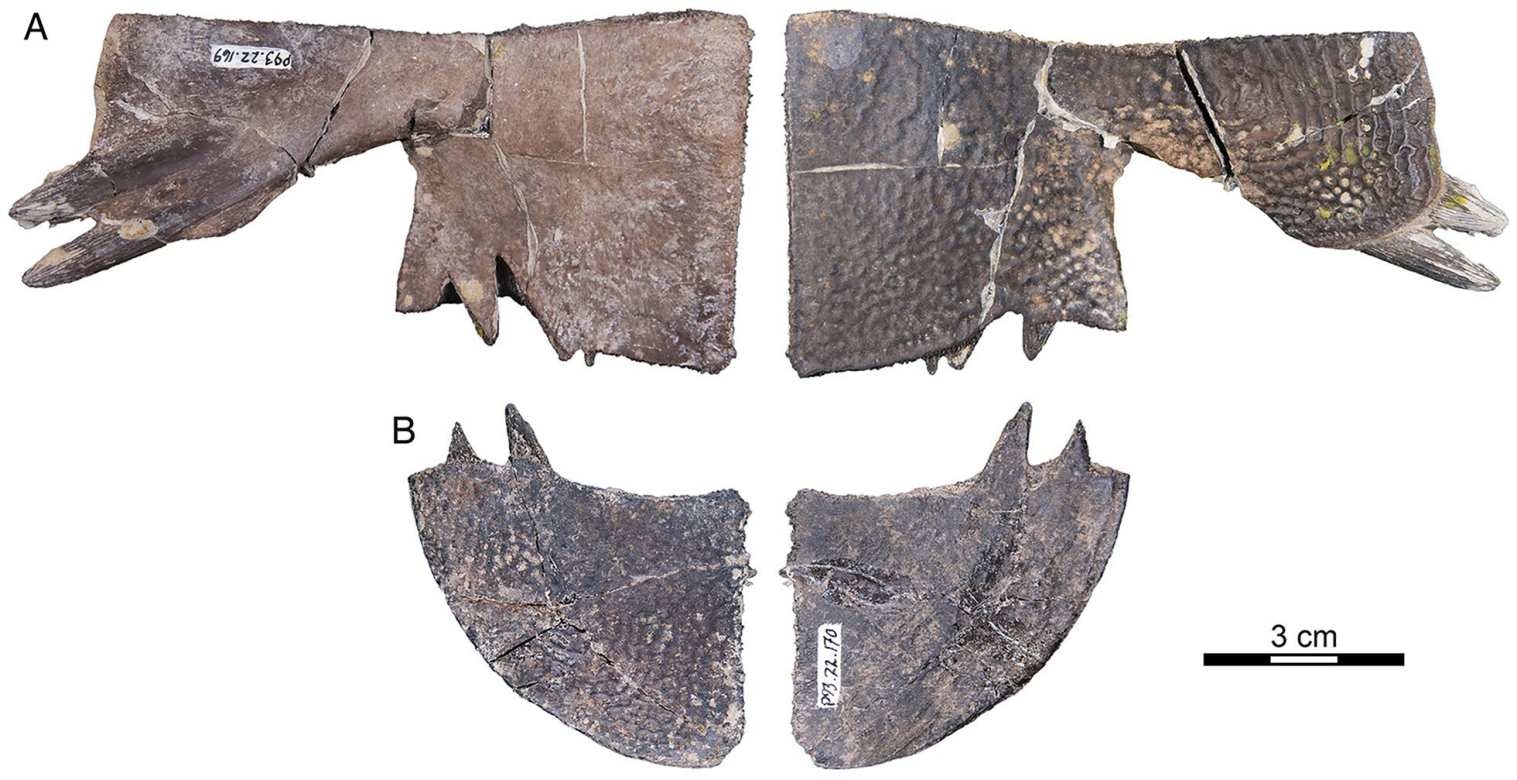
*Hutchemys rememdium*, Wannagan Creek, Bullion Creek Formation, Tiffanian 4, Billings County, North Dakota. **A**, SMM P93.22.169, left hypoplastron in dorsal (left) and ventral (right) views; **B**, SMM P93.22.170, right xiphiplastron in ventral (left) and dorsal (right) views

**Fig. 16:**
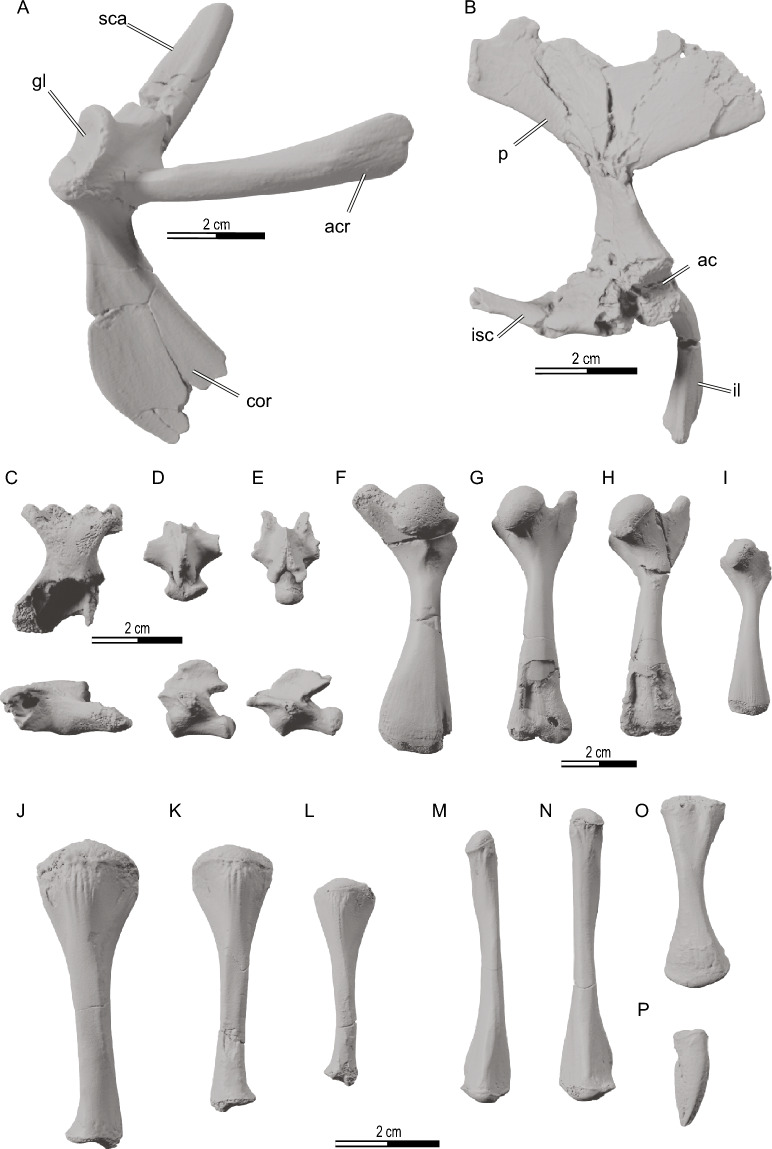
*Hutchemys rememdium*, Ash Coulee Quarry, Sentinel Butte Formation, Tiffanian 4, Billings County, North Dakota. **A**, NDGS 18191, right pectoral girdle; **B**, NDGS 10092, left pelvic girdle; **C**, NDGS 10441, eighth cervical vertebrae; **D**, NDGS 18193, a proximal caudal vertebra; **E**, NDGS 10435, a distal caudal vertebra; **F**, NDGS 11531, a right humerus; **G**, NDGS 10559, a left femur; **H**, NDGS 10361, a left femur; **I**, NDGS 10008, a left femur; **J**, NDGS 10338, a left tibia; **K**, NDGS 10560, a right tibia; **L**, NDGS 10511, a left tibia; **M**, NDGS 10613, a left fibula; **N**, NDGS 10157, a right fibula; **O**, NDGS 18192, a left radius; **P**, NDGS 11481, a claw. *ac* acetabulum, *acr* acromion, *cor* coracoid, *gl* glenoid fossa, *il* illium, *isc* ischium, *p* pubis, *sca* scapula

**Holotype:** YPM PU16795, a nearly complete postcranial skeleton (Joyce et al., 2009).

**Referred Specimens:** *Cranial material:* NDGS 10019, a partial cranium (Figs. 2A, 3, 4, 5; also see 3D model); NDGS 10029, a partial cranium (Fig. 2B, 6; also see 3D model); NDGS 10034, a partial mandible (Fig. 7; also see 3D model); NDGS 10084 (3D Model), NDGS 10329 (3D Model), NDGS 11788 (3D Model), partial mandibles; SMM P77.6.88, a partial dentary (Fig. 8C).

*Shell material:* NDGS 1203, a carapace (Fig. 9A; also see 3D model); NDGS 1758, a plastron lacking epiplastra (Fig. 13A, D); NDGS 9947, an epiplastron (Fig. 14G); NDGS 9975, an epiplastron (Fig. 14L); NDGS 9989, an epiplastron (Fig. 14I); NDGS 10001, a plastron lacking epiplastra (Fig. 14A, D; also see 3D model); NDGS 10023, a plastron lacking epiplastra (Fig. 13B, E); NDGS 10028, a carapace (Fig. 9B); NDGS 10056, a partial plastron (Fig. 14B, E); NDGS 10057, posterior part of carapace (Fig. 10B); NDGS 10071, a partial plastron (Fig. 14C, F; also see 3D model); NDGS 10115, a carapace (Fig. 10C); NDGS 10625, a partial carapace (Fig. 9C; also see 3D model); NDGS 11681, an epiplastron (Fig. 14J); NDGS 11685, an epiplastron (Fig. 14M); NDGS 11689, an epiplastron (Fig. 14H); NDGS 11691, an epiplastron (Fig. 14K); NDGS 11879, a partial carapace (Fig. 9D; also see 3D model); NDGS 12215, posterior half of carapace (Fig. 10A); NDGS 12226, an epiplastron (Fig. 14N); NDGS 18196, a partial plastron (Fig. 13C, F); SMM P77.6.98, a partial left hyoplastron (Fig. 8B); SMM P77.6.99, a left xiphiplastron (Fig. 8D); SMM P77.6.100, a partial costal (Fig. 8A); SMM P91.17.1.1, a partial carapace (Fig. 12A; also see 3D model); SMM P91.17.1.2, a partial plastron (Fig. 12B); SMM P91.17.1.3, a partial plastron (Fig. 12C); SMM P93.22.169, a left hypoplastron (Fig. 15A); SMM P93.22.170, a right xiphiplastron (Fig. 15B); SMM P97.8.26, an associated partial carapace and plastron (Fig. 10; also see 3D model).

*Long bones, girdles, and vertebrae:* NDGS 10008, a left femur (Fig. 16I; also see 3D model); NDGS 10092, a left pelvic girdle (Fig. 16B; also see 3D Model); NDGS 10157, a right fibula (Fig. 16N; also see 3D model); NDGS 10338, a left tibia (Fig. 16J; also see 3D model); NDGS 10361, a left femur (Fig. 16H; also see 3D model); NDGS 10435, a caudal vertebra (Fig. 16E; also see 3D model); NDGS 10441, eighth cervical vertebrae (Fig. 16C; also see 3D model); NDGS 10511, a left tibia (Fig. 16L; also see 3D model); NDGS 10559, a left femur (Fig. 16G; also see 3D model); NDGS 10560, a right tibia (Fig. 16K; also see 3D model); NDGS 10613, a left fibula (Fig. 16M; also see 3D model); NDGS 11481, a claw (Fig. 16P; also see 3D model); NDGS 11531, a right humerus (Fig. 16F; also see 3D model); NDGS 18191, a right pectoral girdle (Fig. 16A; also see 3D Model); NDGS 18192, a left radius (Fig. 16O; also see 3D model); NDGS 18193, a caudal vertebra (Fig. 16D; also see 3D model). All non-shell specimens are available as 3D models at MorphoSource (https://www.morphosource.org/projects/000594396).

**Diagnosis:** *Hutchemys rememdium* can be diagnosed as a representative of *Hutchemys* by the full list of characters listed above. It is differentiated from *Hutchemys sterea* by being approximately twice as large, having a more rounded carapace, a much broader nuchal, a marginal notch between the nuchal and costal I, and shorter posterior costals with a more symmetric split between the surficial and visceral portions of the costals. It is differentiated from *Hutchemys tetanetron* by being about three times larger, a more elongate posterior plastral lobe, and lacking an anal notch. It is differentiated from *Hutchemys arctochelys* by being about two-thirds in size and lacking signs of hyperossification, including elongate costals VIII, surficial sculpturing on the dorsal side of the plastron and the ventral side of the carapace, and a distinct bony flap behind the inguinal process of the hypoplastron (also see Discussion below).

**Range:** Torrejonian and Tiffanian NALMAs (middle and late Paleocene) of Montana, North Dakota, and Wyoming, U.S.A.

## Description

### Skull

*General Comments* The cranium of *Hutchemys rememdium* (Figs. 2, 3, 4, 5, 6), as documented by the new material from North Dakota, has a short triangular snout. The upper temporal emargination is deep, the cheek emargination is weak, the interorbital bar broad, and the postorbital bar narrow. A secondary palate formed by the medial expansion of the maxillae appears to be present. The cranium of *H*. *rememdium* differs greatly from that of *Plastomenus thomasii* and smaller specimens of *Gilmoremys lancensis*, by lacking an elongated, tubular snout, but resembles larger specimens of *G*. *lancensis* by having a triangular snout and broad posterior triturating surfaces. As preserved, NDGS 10019 measures 65.7 mm from the tip of the snout to the base of the basioccipital condyle while NDGS 10029 measures 74.6 mm from the preserved anterior tip of the frontal to the end of the supraoccipital crest.

*Prefrontal* The paired prefrontals of NDGS 10019 are complete, but their descending processes are damaged (Figs. 2, 3, 4). Moreover, both elements seem slightly anteroventrally displaced from their natural position. As the vomer is pushed up into the nasal cavity, the original shape of the fissura ethmoidalis and sulcus vomeri cannot be determined.

The prefrontal forms the anterodorsal part of the snout and the dorsal roof of the apertura narium externa and has a small contribution to the anterodorsal margin of the orbit (Figs. 3, 4). The apertura narium externa is emarginated laterally and therefore has the shape of a truncated heart in anterior view (Fig. 4). This emargination is much shallower than in *Gilmoremys lancensis* and *Plastomenus thomasii*. Medially, the prefrontal contacts its counterpart. Laterally, it superficially contacts the ascending process of the maxilla. Within the nasal cavity, the descending process contacts the maxilla laterally, forms the dorsal part of the suborbital crest (sensu Evers et al., 2023), and laterally frames the fissura ethmoidalis. Mediodorsally, the descending process furthermore contacts the ascending branch of the vomer along a short medial projection. A contact is absent with the palatine, as in all trionychids (Meylan, 1987). On the dorsal skull surface, the prefrontal contacts the frontal posterolaterally. The ventral contact with the frontal is extensive, in that a ventral process of the frontal underlies the prefrontal for three quarters of the prefrontal’s length.

*Frontal* The frontals are complete in NDGS 10019, but only a small fragment of the left frontal is preserved in NDGS 10029 (Figs. 2, 3, 4). The frontals form the interorbital region (Figs. 3, 4). In contrast to *Gilmoremys lancensis* and *Plastomenus thomasii*, they are plesiomorphically developed as a fully unfused pair. Given the size of the skull and the adult nature of the associated shells, we conclude this not to be ontogenetic variation (i.e., indicating a juvenile individual). In dorsal view, only half of the length of the frontal is exposed as a roughly rectangular element. The frontal contacts and underlies the prefrontal anteriorly, the parietal posteriorly, and the postorbital posterolaterally. Along with the prefrontal and postorbital, the frontal forms the dorsal margin of the orbit. Medially the frontals contact each other. Anterior to the crista cranii (Fig. 4C), the frontals jointly form a triangular median process that projects below much of the prefrontal. This anterior process was explicitly reported for *P*. *thomasii*, but our study of other trionychids suggests it is universally present. Within the orbit, the contact of the frontal with the prefrontal is deeply interdigitated. On the dorsal skull surface, the frontal contacts the parietal along a deeply interdigitated suture (Fig. 3). In addition, the posterior third of the frontal is underlapped by an anteriorly projecting process of the parietal, which mediates between the crista cranii and the descending process of the parietal. In the interorbital region, the frontal forms the crista cranii with a minor posterior contribution of the parietal. The ridges of the crista cranii are low, but well-defined. Even though the sulcus olfactorius is just as shallow as in *G*. *lancensis* and *P*. *thomasii*, the ridge that defines the crista cranii is less distinct.

*Parietal* The parietals are present as large, paired elements that make up most of the posterior part of the skull roof and contribute to the lateral wall of the braincase and the processus trochlearis oticum (Figs. 2, 3, 4, 5D, 6A). They are better preserved in NDGS 10019, where they are complete, but fractured. The parietals are particularly fractured anteriorly, at the level of the processus trochlearis oticum, the secondary wall of the braincase, along the lateral processes, and at the posterior tip of the parietals. In NDGS 10029, their anterior and ventral portion, as well as their posterior tip are missing. Only the left parietal partly preserves its lateral portion.

The parietal contacts the frontal and postorbital anteriorly, the palatine and epipterygoid ventrally, the prootic and supraoccipital posteriorly, and its counterpart medially. Contacts with the jugal and pterygoid are absent. On the dorsal skull roof, the parietal is flat and smooth. Anteriorly, the parietal contacts the fontal and postorbital along sutures that are not just deeply interdigitated along the surface, but also at depth. Each parietal forms a distinct anterior process that broadly underlies each frontal, reaches the level of the orbit, and forms the most posterior aspects of the crista cranii. A contact between the jugal and the parietal is prevented by the relatively large postorbital (Fig. 3). This differs from the condition present in *Gilmoremys lancensis* and *Plastomenus thomasii*, in which the postorbitals are reduced or absent, respectively. This contact is known to be variable among other trionychids as well (Meylan, 1987). In contrast to *P*. *thomasii* and at least one individual of *G*. *lancensis*, the parietal does not contribute to the orbit margin.

The processus inferior parietalis broadly contacts the ascending process of the palatine anteriorly and the epipterygoid posteriorly and forms the secondary wall of the braincase (Fig. 5D). The dorsal margin of the right foramen nervi trigemini of NDGS 10019 shows clear signs of damage, while the left one seems to be intact at least in part. We interpret a circular notch on the ventral side of the parietal of this specimen to represent a genuine contribution of the parietal to the anterodorsal border of the foramen nervi trigemini (Fig. 5D). This contrasts with both *P*. *thomasii* and *G*. *lancensis*, where the parietal is excluded from the foramen nervi trigemini by an extensive contact between the epipterygoid and prootic. However, there is variation in this character among other trionychids (Meylan, 1987).

Within the upper temporal fossa, the parietal broadly overlaps the prootic. The contact is supported at depth by numerous fine interdigitations. The parietal forms nearly a quarter of the processus trochlearis oticum (Fig. 3). Farther posteriorly, the parietal sits on top of the supraoccipital. Similar to the contact with the prootic, the parietal contact with the supraoccipital is stabilized internally by numerous interdigitations. Towards the posterior tip of the skull, the parietal thins into a blade of bone underlain by the supraoccipital crest. The transition between the dorsal and the posterolateral surfaces of the parietal is marked by a steplike ridge. The parietals jointly form the anterior half of the vault-like cavum cranii.

*Postorbital* In NDGS 10019, both postorbitals are present and complete, while in NDGS 10029, only the ventral portion is preserved (Figs. 2, 3, 4).

In dorsal view, the postorbital is overall triangular in shape (Fig. 3). Lateroventrally, it contacts the jugal along a smooth contact on the skull surface, but an interdigitated contact within the orbit. The postorbital forms a broadly concave contact with the frontal anteromedially and a deeply interdigitated suture with the parietal posteromedially. The postorbital is greatly expanded ventrally and almost reaches the floor of the orbit.

The postorbital of trionychids varies greatly in size (Meylan, 1987) and is even reduced to absent in *Gilmoremys lancensis* and *Plastomenus thomasii*, respectively. In contrast, the postorbital in *Hutchemys rememdium* is well-developed. The postorbital forms the posterodorsal margin of the orbit and forms the majority of the dorsal portion of the postorbital bar. The postorbital fully prevents the contact between the jugal and parietal, not only on the skull surface and within the temporal fossa, but also within the skull, as confirmed by the available µCT slices. Thus, the postorbital participates in the formation of the anterior part of the temporal emargination.

*Jugal* The jugals are both preserved in NDGS 10019 (Figs. 2A, 3, 4). The left jugal of this specimen lacks its posteroventral process, while the right jugal is only missing the most posterior tip of this process. In NDGS 10029, the left jugal is preserved with the posterior process intact, while the median process and the portion participating in the floor of the orbit are missing. The right jugal is missing in this specimen (Figs. 2B).

The jugal has a broad V-shaped contact with the maxilla anteriorly, the postorbital dorsally and medially, the palatine medially, and the quadratojugal posteriorly (Figs. 3, 4). A minor posteromedial contact with the pterygoid may have been present as well, but is obscured by damage. Anteriorly the jugal extensively contacts the maxilla and participates in the posteroventral margin of the orbit. Just anterior to the cheek emargination, the jugal is laterally overlapped by a triangular process of the maxilla. In addition, the jugal buttresses the posterior portions of the triturating surface formed by the maxilla. In this area, a posteroventral process of the jugal contacts the palatine medially at the level of the “foramen palatinum anterius” (see Palatine below, Fig. 5C). This contact of the jugal and palatine continues posteriorly, is underlaid by the maxilla, and forms the posterior margin of the postorbital fenestra and the anterior margin of the lower temporal fossa. It is not clear if the jugal and pterygoid contact one another as this area is damaged on both sides of NDGS 10019, but this contact is usually present, albeit greatly reduced, in some trionychids (Gaffney, 1979). The jugal contacts the postorbital dorsally and the two bones jointly form the postorbital bar (Figs. 3, 4). The jugal forms the majority of the anterior portion of the temporal arch with its ascending and posterior processes. A jugal-parietal contact, which is either surficial or internally present in many trionychids, is fully prevented by the large postorbital. Posterolaterally, the jugal forms an anteroposteriorly elongate, sheetlike process that delimits the temporal fossa and broadly covers the quadratojugal laterally. The jugal hereby partially encapsulates the anterior most end of the quadratojugal. Contrary to *Plastomenus thomasii* and some specimens of *Gilmoremys lancensis*, there is no jugal-squamosal contact. Indeed, the bones are broadly separated from one another in NDGS 10029. This is the more common condition within extant trionychids with the squamosal-jugal only occasionally being in contact in some trionychines (Meylan, 1987) and the plastomenids *P*. *thomasii* and *G*. *lancensis*.

*Quadratojugal* In NDGS 10019, both quadratojugals are damaged (Figs. 2A, 3, 4, 5D). Only the anteriormost part of the dorsoposterior process remains on the left side. The right quadratojugal is missing the ends of both its posterior processes, while the anterior process is damaged. In NDGS 10029, only the left quadratojugal remains. Its anterior portion is well-preserved but the posterior processes are missing (Fig. 2B).

The fragmentary remains of the quadratojugal jointly suggest that this bone is a three-pronged element that makes up the posterior portion of the temporal arch and outlines the anterior margin of the cavum tympani, as in all trionychids (Fig. 3). The quadratojugal contacts the jugal anteriorly, the quadrate posteriorly, and the squamosal posterodorsally. Anteriorly, the tip of the thin anterior process of the quadratojugal is encapsulated by the jugal. Most of the length of the quadratojugal is concealed by the jugal in lateral view. The quadratojugal is exposed medially for about half of the length of the arch. The two posterior processes contact the quadrate posteromedially along an arched suture that outlines the margin of the cavum tympani without apparently contributing to it. In NDGS 10019, the anterior process of the squamosal is missing, but a former contact with the quadratojugal is made visible by an articular facet. In NDGS 10029, the contact is preserved on the left side, but the area is too damaged to document specific details. The quadratojugal forms the medial margin of the upper temporal fossa and prevents the contact between the jugal and squamosal (see Jugal above).

*Squamosal* In NDGS 10019, only the right squamosal is preserved, though incomplete, heavily fractured, and rotated from its likely natural position (Figs. 2A, 3, 4). In NDGS 10029, the anterior part of the left squamosal is preserved at its contact with the quadratojugal while the posterior part of the right squamosal is preserved behind the antrum postoticum (Figs. 2B, 6A). The portion of this bone contributing to the antrum postoticum is collapsed into the antrum, but the posterior process of the squamosal is intact.

The posterior half of the squamosal forms a conical cavity with the quadrate (best visible in the models). This hollow cavity, the antrum postoticum, is formed dorsolaterally by the squamosal and ventromedially by the quadrate and anteriorly confluent with the cavum tympani. The squamosal ventrally contacts the paroccipital process of the opisthotic along an extended contact (Fig. 6A). The flattened posterior process of the squamosal is elongated and decorated by a vertical ridge, which is slightly sigmoidal in dorsal view. The posterior process of the squamosal does not reach the level of the posterior end of the supraoccipital crest. The poorly preserved anterior half of the squamosal is sheet-like and contacts the quadratojugal lateral to the processus trochlearis oticum. This contact is visible in NDGS 10029 despite damage to this area of the skull and documented by an articular scar in NDGS 10019. Unlike *Plastomenus thomasii* and some specimens of *Gilmoremys lancensis*, the squamosal does not contact the jugal. While the greatest part of the anterior portion of the squamosals are missing, the remaining articular scars along the dorsal margin of the quadrates suggest an extensive and complex contact. As in other trionychids, the squamosal likely covered the quadrate to roof the cavum tympani and antrum postoticum.

*Premaxilla* The anterior tip of the snout is only preserved in NDGS 10019, but the fused premaxillae are not preserved (Fig. 4). Instead, a gap is apparent between the anterior tips of the maxillae with a floating piece of bone that could be interpreted as the premaxilla. We cannot determine with confidence if the maxillae are damaged in this region and therefore cannot clarify if premaxillae were once present but taphonomically lost, or absent in the first place.

*Maxilla* The two maxillae are overall well-preserved though heavily fractured in the palatal region of NDGS 10019 (Figs. 2, 3, 4, 5C). While much of the left palatal region is missing, numerous fragments cannot be attributed to any particular bone with confidence. Anteriorly, the area of the foramen intermaxillary is badly preserved and the shape of this foramen cannot be determined. When viewed from the anterior, it is apparent that both maxillae rotated along a sagittal axis located just below the eye (Fig. 4). As a result, the ascending processes are now pointing laterally, while the enlarged triturating surfaces rotated into the interorbital space, thereby creating the Illusion of an extremely vaulted secondary palate. The vaulted appearance is further amplified by fractures, along which the median portion of the secondary palate is even further moved dorsally.

The maxilla forms the ventral and lateral aspects of the snout. Anteriorly, it converges with its counterpart, but this is exaggerated by the taphonomic rotation mentioned above. The triangular outline of the triturating surfaces (Figs. 3, 4), however, is supported by the shape of the dentary (see Dentary below). Anterodorsally, the maxilla contacts the prefrontal along a triangular ascending process, forms the lateral rim of the apertura narium externa, and contributes to the anterior margin of the orbit. The mid-section of the maxilla floors much of the orbit and contacts the jugal (Fig. 3). The maxilla generally underlies the jugal, but the contact is further stabilized by a triangular posterior process of the maxilla that interfingers with two anterior processes formed by the jugal. A suborbital crest is weakly developed between the nasal and orbital cavities. This ridge is much more distinct in *Plastomenus thomasii*. Although the posterior contacts are obscured by damage and the uncertain assignment of various bony fragments, it is nevertheless clear that the maxilla farther posteriorly forms the posterior end of the triturating surface and contacts the palatine ventrally and medially and the pterygoid posteriorly.

The triturating surface is laterally delimited by a high, vascularized labial ridge (Fig. 4). Multiple foramina are present on the keel of the ridge and its lateral and medial sides. These foramina connect to a network of canals that exit dorsally through foramen alveolare superius, which is located just inside the nasal cavity, and the foramen supramaxillare, which is located within the floor of the orbit just medial to the anterior jugal process (Fig. 5C; Albrecht, 1967). The two foramina are associated with distinct posteriorly leading grooves, indicating the former path of the associated arteries. This differs from the condition in *P*. *thomasii*, where only a fenestra is present without a groove at the level of the foramen alveolare superius.

As noted above, the median portions of the palate appear to have been taphonomically pushed up dorsally into the interorbital space and rotated upwards. As a result, the area appears more vaulted than in life. Much as in *Gilmoremys lancensis* and *P*. *thomasii*, the maxillae nevertheless seem to have formed broad triturating surfaces and an extensive secondary palate. In contrast to *P*. *thomasii* and juvenile* G*. *lancensis*, but much as in the largest known *G*. *lancensis*, the palate is broadly expanded posteriorly to form the shape of an equilateral triangle. The presence of a clear surficial facet along the posterior parts of the vomer in combination with medial facets along the anterior portions of the maxilla suggests that the maxillae met their counterpart along the midline in the anterior parts of the palate, but not posteriorly. Although the medial region of the palate is pushed up into the nasal cavity, the maxilla gently curves dorsally towards the midline and to form a relatively broad median palatal groove, as in *P*. *thomasii* and *G*. *lancensis*. There is no accessory ridge on the triturating surface, as is present in *G*. *lancensis*. The maxilla minorly contributes to the lateral border of the foramen palatinum posterius, but not to the canalis intrapalatinus. This is best seen in the 3D models.

*Vomer* The vomer is a single, median element only preserved in NDGS 10019 (Fig. 4C, D). Due to taphonomic damage to the palatal region of the skull, the posterior and anterior most ends are missing and the vomer is pushed up into the nasal cavity and minorly displaced posteriorly. The tips of the ascending processes of the vomer for articulation with the prefrontal are present but separated from the main body of the vomer by a break. The base of the ascending process is only preserved on the right side in articulation with the rest of the bone.

The vomer contacts the maxilla lateroventrally, the palatine posteriorly, and the prefrontal dorsally (Fig. 3, 4C, D). The sheet-like main body of the vomer dorsally covers the median sutures and gaps between the maxillae (see Maxilla above). The vomer otherwise forms a flat, ventral facet located behind the maxillae that is flush with the triturating surfaces and may have even contributed to them. Along the midline, the vomer rises to form a narrow ridge that is capped by a shallow sulcus vomeri. The dorsolateral contact with the palatine is not preserved but was likely present as in all trionychids. Anteriorly, the vomer contacts the descending processes of the prefrontals along a pair of ascending processes. It is not clear if the vomer anteriorly reaches the intermaxillary foramen as this region is poorly preserved. In ventral view (Fig. 4C, D), the vomer was exposed in the posterior region of the palate as indicated by a flat ventral surface posterior to the median contact of the maxillae.

*Palatine* The palatines are paired, medial elements on the posterior part of the palate, only preserved in NDGS 10019 (Figs. 4C, D, 5D). The anterior part of both palatines is heavily damaged and the medial portion that should form the roof of the narial canals is lost.

The palatine is a complex bone participating in the posterior part of the palate, flooring the anterior part of the brain case, and contributing to the secondary wall of the braincase (Figs. 4C, D, 5D). It contacts the maxilla anteriorly, the jugal anterodorsolaterally, the pterygoid lateroventrally, the parabasisphenoid ventrally, the descending process of the parietal and epipterygoid dorsally along an ascending process, the vomer anterodorsally, and its counterpart along the midline. The palatine and parietal are tightly interlocked. The ascending process of the palatine reaches its greatest height at the anterior part of the secondary braincase wall and slopes downwards posteriorly until reaching the epipterygoid anterior to the foramen nervi trigemini (Fig. 5D). Along the medial side of the parietal contact, the ascending process of the palatine remains relatively low and the wall is mostly formed by the parietal. In *Hutchemys rememdium* the secondary wall of the braincase is thick compared to that of *Plastomenus thomasii*. Anterior to the ascending process, the palatine forms an anteroposterior process that is continuous with the secondary wall of the braincase and inserts into the posterodorsal margin of the maxilla. The medial part of the palatine is missing on both sides. The more lateral remainders of this bone nevertheless form the lateral edges of the choana (Fig. 4C, D). In the floor of the orbit, lateral to the ascending process, the palatine contacts the jugal laterally and above the maxilla. The contact is thickened to form the posterior margin of the postorbital fenestra and the anterior margin of the lower temporal fossa. Anterolateral to the base of the secondary wall of the braincase, the palatine encloses most of the canalis palatinus major (sensu Ogushi, 1911), the remainder being formed by the maxilla. The canal connects the classic “foramen palatinum posterius” of turtles, which is located laterally to the internal choanae, with an anterior opening that is located in the posterolateral floor of the orbit and that we here term the “foramen palatinum anterius” (Fig. 5C). This canal holds the inframaxillary artery (Albrecht, 1967). In many trionychids, secondary canals branch off the canalis palatinus major to exit medial to the foramen palatinum posterius in the roof of the palate (Albrecht, 1967). These canals, termed canalis palatinus minor by Ogushi (1911) but grouped with the canalis palatinus major into the interpalatine canal by Albrecht (1967), are not apparent in *H*. *rememdium*, but it is unclear if this is genuine or the result of damage. In contrast to *Gilmoremys Lancensis* and *Plastomenus Thomasii*, which exhibit two or more small foramina palatinum posterius, we can only observe a single, small foramen in NDGS 10019. Unlike *P*. *thomasii*, the foramen palatinum anterius is not aligned with the foramen supramaxillare but more so with the foramen alveolare superius. In its posterior portion, the palatine encloses the canalis nervus vidianus (Rollot et al., 2021). This canal contains the anterior portion of the vidian nerve. It opens posteriorly into the sulcus cavernosus and anteriorly into the canalis palatinus major through the foramen anterius canalis nervus vidianus. Posterior to the vidian canal, the palatine forms the anterior portion of sulcus cavernosus, which is otherwise formed by the pterygoid.

The contact of the palatine with the vomer is not preserved, because the posterior end of the vomer and the anterior parts of the palatines are damaged. It is likely, however, that the anterior part of the palatine overlapped the posterior portion of the vomer, as in most trionychids.

*Pterygoid* The pterygoids of NDGS 10019 are overall complete but deformed by numerous breaks (Figs. 3, 4, 5A, B, D, 6A). A particularly large break, which also affects the parabasisphenoid, crosses the skull in a reversed V-shape. NDGS 10029 is badly crushed and the pterygoids are broken into multiple pieces. As a result, most features and contacts are obscured.

The pterygoid contacts the maxilla and jugal anterolaterally, the palatine anteromedially, the parabasisphenoid medially, and the basioccipital and exoccipital posteromedially. As in the rest of the skull, most contacts are deeply interdigitated. Dorsally, the pterygoid contacts the epipterygoid, the prootic, the quadrate, and the opisthotic (Figs. 3, 4). Although the median region of the skull is not preserved in the available specimens, the reduced surficial contribution of the parabasisphenoid to the ventral flooring of the skull in NDGS 10019 strongly suggests that the pterygoids contacted one another along the midline in this specimen. At the same time, the parabasisphenoid broadly roofs the midline contact of the pterygoids. Among extant trionychids, this unusual combination of characteristics is only seen in *Rafetus euphraticus* (Meylan, 1987; NMW 132). The pterygoid forms an anterior process that contacts the maxilla posterior to the triturating surfaces, best preserved on the left side of NDGS 10019 (Fig. 4). This process resembles that of many extant trionychids by being short and broad, whereas the process of *Gilmoremys lancensis* and *Plastomenus thomasii* are notably elongate. Medial to this contact, the pterygoid contacts the palatine extensively along an oblique contact. As in all trionychids, *Hutchemys rememdium* does not form a laterally protruding external process of the pterygoid. Instead, just posterior to the contact with the maxilla, the lateral margin of the pterygoid forms a vertical plate just above a lateral constriction. Posterior to this constriction, the pterygoid laterally thins and widens into a smooth, plate-like sheet of bone, which we here term the pterygoid flange (sensu Gaffney et al., 2006 for pleurodires). Although this area was diagenetically flattened in NDGS 10019, this part of the pterygoids, combined with the parabasisphenoid, likely formed a low, broad convexity. At the level of the posterior margin of the foramen nervi trigemini, the pterygoid flange is at its widest. Posterior to this point, the pterygoid flange forms a second constriction, along which the margin is notably thickened. At the level of the articular condyles, the ridge of the flange splits into two branches, of which the lateral leads to the articular condyles, while the medial leads to the “infolding ridge” of the quadrate (sensu Evers et al., 2023; see below). The two branches jointly define a triangular pterygoid fossa, which appears to be relatively shallow in NDGS 10019, but much more distinct in NDGS 10029. The pterygoid floors the canalis cavernosus (Fig. 5A, B), the cavum acustico-jugulare, and the recessus scalae tympani. The pterygoid completely encloses the posterior portion of the canalis caroticus internus. This canal enters the skull posteriorly through the foramen posterius canalis caroticus internus, at the level of the parabasisphenoid-basioccipital suture (Fig. 5A).

The ascending process of the pterygoid, the crista pterygoidea (Gaffney, 1972), is low and contacts the epipterygoid anteriorly and the quadrate posteriorly. The crista forms the posteroventral margin of the foramen nervi trigemini (Fig. 5D) and laterally forms the wall of the canalis cavernosus and the posterior part of the sulcus cavernosus. The contribution of the pterygoid to the foramen nervi trigemini is variable within extant trionychids (Meylan, 1987). In *G*. *lancensis* and *P*. *thomasii*, this contact is absent (Evers et al., 2023; Joyce & Lyson, 2011; Joyce et al., 2016). The internal carotid artery is dorsally exposed within the cavum acustico jugulare (Figs. 5A, B), as mentioned by Rollot et al. (2021) for *Amyda cartilaginea*, *Chitra indica*, *Pelodiscus sinensis*, and *Cycloderma frenatum*. The canalis caroticus lateralis is reduced to a foramen in *H*. *rememdium* of which the pterygoid forms the lateral margins. This foramen opens to the sulcus cavernosus, lateral to the canalis caroticus basisphenoidalis (sensu Rollot et al., 2021).

The posterior processes of all available pterygoids are damaged, but the relatively intact basioccipital of NDGS 10029 suggests an elongate posteromedial contact between these two bones (Fig. 6A). NDGS 10029 tentatively suggests the presence of a ventral contact with the exoccipital. The pterygoid certainly formed the ventral margin of the fenestra postotica and the foramen jugulare posterius, but damage once again precludes assessing if these two structures were separated by the pterygoid, exoccipital, or opisthotic, as seen in *G*. *lancensis* and *Plastomenus thomasii*.

*Epipterygoid* The epipterygoids are only preserved in NDGS 10019 (Figs. 3, 5D). As in most trionychids, the epipterygoid is a flat, sub-triangular element. It has a short contact with the palatine anteriorly, a more elongate contact with the parietal anterodorsally, forms the ventral margin of the elongate trigeminal foramen, and broadly contacts the crista pterygoidea ventrally and medially (Fig. 5D). An epipterygoid contact with the prootic anterior to the trigeminal foramen is well-developed in *Gilmoremys lancensis* and *Plastomenus thomasii*, but absent in *Hutchemys rememdium*.

*Quadrate* The paired quadrates are both damaged in the two available specimens (Figs. 2, 3, 4, 5D, 6A). In combination, however, the two specimens document all aspects of this element.

Anterior to the cavum tympani, the quadrate forms a broad inverse C-shaped contact with the quadratojugal. The quadrate medially contacts the prootic for all of the prootic’s length. Posteromedially the quadrate contacts the opisthotic (Figs. 3, 4). The dorsolateral contact with the squamosal is only partially preserved in NDGS 10029, but the articular scars on the quadrate suggest this contact was relatively extensive. The articular scars of the quadrate with the squamosal within the upper temporal fossa suggest that the quadrate was only dorsally exposed as a thin band of bone between the prootic and opisthotic medially and the squamosal laterally. Nonetheless, the quadrate contributes to the lateral quarter of the processus trochlearis oticum, which is otherwise formed by the prootic and parietal (Fig. 3).

The quadrate forms the lateral margin of the stapedial foramen. The quadrate furthermore forms the lateral side of canalis stapedio-temporalis, which opens posteriorly into the aditus canalis stapedio temporalis in the roof of the cavum acustico jugulare. In this area, the quadrate also forms the lateral wall of the canalis cavernosus. At the level of the sulcus cavernosus, the quadrate forms the surficial margin of the foramen nervi trigemini posteriorly (Fig. 5D).

The quadrate forms the anteroposteriorly elongated, triangular shaped cavum tympani (best viewed in 3D models). Posterior to the cavum tympani the quadrate contributes to the median wall of the elongated, conical antrum postoticum, which is fully confluent with the cavum tympani. The enclosed incisura columella auris is teardrop-shaped and posteroventrally connected to a low ridge that does not reach the edge of the cavum tympani.

The articular condyle of the quadrate is best preserved in NDGS 10029 (Fig. 6A). All trionychids possess the “infolding ridge” (Evers et al., 2023) on the posterior surface of the quadrate dorsal to the articular condyle. In *Hutchemys rememdium*, this ridge is prominent, rises from the laterodorsal edge of the condyle, runs lateromedially towards the pterygoid, ventrally borders the Eustachian notch, and connects with the pterygoid. A concavity is created between the ridge and the articular condyle (Fig. 6A). The infolding ridge of *Plastomenus thomasii* is more ventrally located and defines a less prominent cavity.

*Prootic* The prootics are both preserved and relatively intact in NDGS 10019 while they are both ventrally and anteriorly damaged in NDGS 10029 (Figs. 2, 3, 4C, D, 5D). They are paired bones that contribute to the braincase, temporal fossa, and the roof of the otic capsule. Anteriorly, the prootic is extensively overlapped by the parietal. It contacts the supraoccipital medially, the opisthotic posteriorly, the quadrate laterally, the pterygoid ventrally, and the parabasisphenoid ventromedially (Figs. 3, 4B, D, 5D).

Within the temporal fossa, the prootic forms the largest portion of the concave processus trochlearis oticum, about half of its width. The prootic contributes to the dorsal margin of the foramen nervi trigemini (Fig. 5D) and forms the medial part of the foramen stapedio-temporale and the canalis stapedio-temporalis, which opens ventrally into the cavum acustico-jugulare and dorsally into the floor of the temporal fossa. The prootic also forms the anterolateral wall of the cavum acustico-jugulare.

The prootic forms the anterior part of the cavum labyrinthicum and the lateral wall of the cavum cranii (observable in the 3D models). The anterior semicircular canal is fully enclosed by bone between the prootic and supraoccipital. The prootic entry is located towards the anterior tip of the triangular cavum labyrinthicum. Although we cannot exclude damage, the horizontal semicircular canal of both prootics of both specimens does not seem to be enclosed by bone. Instead, a low, broad sulcus is apparent between the prootic and opisthotic in the roof of the cavum labyrinthicum. This differs from *Plastomenus thomasii*, which exhibits an enclosed horizontal semicircular canal. The prootic otherwise forms the anterior margin of fenestra ovalis. This fenestra, formed posteriorly by the opisthotic, separates the medial cavum labyrinthicum from the lateral cavum acustico-jugulare. This fenestra is damaged in both specimens but appears to not have been enclosed by the prootic or opisthotic ventrally. It is plausible that the fenestra ovalis was unossified ventrally, as in other trionychids, including *P*. *thomasii*. Alongside the quadrate and pterygoid, the prootic contributes to the dorsal and medial wall of canalis cavernosus.

The foramina nervi acustici and the foramen nervi facialis are visible within the deep fossa acustico-facialis, which houses the ganglion vestibulare. At least two foramina nervi acustici can be observed within the fossa of the left prootic of NDGS 10029. They indicate the passageway of the different branches of the acoustic nerve (VIII) from the cavum cranii to the cavum labyrinthicum (Rollot et al., 2021). The geniculate ganglion (Fig. 5A, B) is located within the prootic. The canalis nervus facialis, which houses the facial nerve, therefore crosses only about two-thirds of the distance from the fossa acustico-facialis towards the canalis cavernosus. The remaining distance from the ganglion to the canalis cavernosus is crossed by the canalis nervus hyomandibularis, which holds the hyomandibular branch of the facial nerve (Rollot et al., 2021). As the geniculate ganglion is also located close to the canalis caroticus internus, the canalis pro ramo nervi vidiani (sensu Rollot et al., 2021) is short, though likely mostly located within the pterygoid.

The prootic of *Hutchemys rememdium* does not reach as far anteriorly in the area of the foramen nervi trigemini as in *Plastomenus thomasii* and *G*. *lancensis*, where the parietal is excluded from the foramen nervi trigemini by a contact of the prootic with the epipterygoid. Instead, the prootic of *H*. *rememdium* contacts the parietal along the dorsal margin of the foramen nervi trigemini (Fig. 5D). The shape of the foramen nervi trigemini is best preserved on the left side of NDGS 10019 where it is anteroposteriorly elongated and about three times longer than high. This differs from the higher, but shorter trigeminal foramina of *G*. *lancensis* and *P*. *thomasii*.

*Opisthotic* The two opisthotics are present but damaged in NDGS 10019 (Figs. 2A, 3, 4A, 4C). The paroccipital processes and the ventral part of this bone of this specimen are missing, but the processus interfenestralis are preserved, if damaged. In NDGS 10029, both opisthotics are preserved with the dorsal margin and paroccipital processes overall intact, but the ventral portion is crushed (Figs. 2B, 6A).

The opisthotic bone forms the posterolateral portion of the otic capsule. The opisthotic contacts the exoccipital posteromedially, the pterygoid ventrally via the processus interfenestralis, the quadrate laterally, the supraoccipital medially, and the prootic anteriorly (Figs. 3, 4). The opisthotic forms the posterolateral roof of the cavum labyrinthicum. The portion of the canalis semicircularis horizontalis formed by the opisthotic is enclosed in bone in both specimens. The opisthotic portion of the canalis semicircularis posterior, however, is enclosed in NDGS 10029, but remains open on both sides of the skull of NDGS 10019. A minor roughening apparent to the roof of the cavum labyrinthicum might suggest that this is the result of damage. The descending process of the opisthotic, called the processus interfenestralis, participates in the posterior margin of the fenestra ovalis, which separates the medial cavum labyrinthicum from the lateral cavum acustico jugulare. Only the dorsal aspects of the fenestra perilymphatica, which connects the cavum labyrinthicum with the recessus scalae tympani, are preserved between the processus interfenestralis and remnants of a more medial descending process of the opisthotic. These structures are best seen on the left side of the models of NDGS 10019. The opisthotic forms the anterior margin of the unossified hiatus acusticus. This gap connects the cavum labyrinthicum to the cavum cranii. The foramen jugulare anterius is formed medially and dorsally by the opisthotic as a large opening connecting the recessus scalae tympani and cavum cranii. Posteriorly, the opisthotic sends a medial process between the supraoccipital and the exoccipital. In this area, the opisthotic clasps the supraoccipital with a thin layer of bone dorsally and a thicker process ventrally. The opisthotic forms a curled paraoccipital process that underlies the median margin of the quadrate and the squamosal. The part of the paraoccipital process underlying the squamosal forms a medial ridge continuous with the dorsal margin of the fenestra postotica. The contact with the squamosal extends along the full length of the paraoccipital process. The opisthotic forms the dorsal and medial border of the fenestra postotica and the lateral border of foramen jugulare posterius. The two foramina are fully confluent, which contrasts with *G*. *lancensis* and *P*. *thomasii*, where they are separated by a bar formed by the opisthotic and exoccipital.

*Supraoccipital* In NDGS 10019, the posterior parts of the supraoccipital crest are missing, while the remainder of the bone is deformed by small breaks (Fig. 2A, 3, 4A, C). In NDGS 10029, the braincase is crushed, but the supraoccipital crest is entirely preserved despite several breaks (Figs. 2B, 6A).

The supraoccipital is a large element roofing the dorsal part of the braincase. Anteriorly and dorsally, the supraoccipital contacts the parietals and laterally contacts the prootic, opisthotic, and exoccipital along symmetrical lateral processes (Figs. 3, 4C, D). The main body of the supraoccipital forms the posterior part of the cavum cranii and the dorsal part of the large foramen magnum. Anteriorly, the supraoccipital broadly underlies the parietal but overlaps the prootic. Dorsally, the supraoccipital rises to form a crest. The crest continues posteriorly beyond the posterior edge of the occipital condyle to form the crista supraoccipitalis. The crest has an upside-down T-shape in cross-section, as in all trionychids, due to the presence of a posteriorly tapering horizontal plate at its base. The junction points of the supraoccipital, prootic, and opisthotic are marked by wide, non-interdigitated sutures, which also marks the thickest point of the supraoccipital (Fig. 3). Farther posteriorly, the supraoccipital forms a laminar triangular process that intrudes laterally into the posterior portion of the opisthotic. This process is partially visible on the posterior skull surface. A ridge emerges from the surface of this process that continues medially to form the horizontal plate of the supraoccipital crest (Fig. 6A).

Within the skull, the supraoccipital roofs the medial third of the cavum labyrinthicum. At the level of the prootic-opisthotic suture, the supraoccipital forms a deep notch, which represents the dorsal margin of the unossified hiatus acusticus. The posteromedial portion of the anterior semicircular canal, which is formed by the supraoccipital, is fully enclosed by bone. The anteromedial portion of the posterior semicircular canal, by contrast, is ventrally open. This differs from *Plastomenus thomasii*, which has a fully enclosed posterior semicircular canal (Evers et al., 2023). The common crus is located just above the hiatus acusticus. A notch above the posterior opening of the anterior semicircular canal connecting the cavum labyrinthicum with the cavum cranii is preserved on the left side of the skull of NDGS 10019 and may be the only preserved traces of the foramen aquaducti vestibuli. This differs from the condition in *P*. *thomasii* where the foramen aquaducti vestibuli is completely formed within the supraoccipital.

*Exoccipital* The exoccipitals are present in both skulls. They are badly damaged in NDGS 10019 (Figs. 3, 4A, C) but well-preserved in NDGS 10029, where the occipital condyle is complete and the right exoccipital is mostly intact (Fig. 6).

The exoccipital forms the lateral and ventral margins of the large foramen magnum as well as the medial margin of the foramen jugulare posterius, which is confluent with the fenestra postotica. The exoccipital contacts the supraoccipital dorsally, the opisthotic dorsolaterally, the pterygoid laterally, and the basioccipital ventrally. Combined, the exoccipitals form the majority of the occipital condyle. The exoccipital share a long median contact with each other from just behind the foramen magnum to the posterior tip of the occipital condyle, much as in *Gilmoremys lancensis* and *Plastomenus thomasii*. The right exoccipital of NDGS 10029 exhibits three aligned foramina nervi hypoglossi that are covered from ventral view by the basioccipital tubercles (Fig. 6B). The most posterior and dorsal foramen is completely formed by the exoccipital while the next one is mostly formed by the exoccipital with a minor, ventral contribution from the basioccipital. Finally, the anteriormost foramen is largely formed by the basioccipital and the exoccipital only forms a small, internal section of the foramen.

*Basioccipital* The basioccipital is greatly damaged in NDGS 10019 (Figs. 3B, D, 4A, C), but is well-preserved in NDGS 10029 (Fig. 6).

The basioccipital is a single, median element flooring the posterior portion of cavum cranii and forming the ventromedial third of the occipital condyle (Fig. 6). The basioccipital contacts the parabasisphenoid anteriorly, the pterygoid anterolaterally and the exoccipital posterodorsally. As the ventral part of the opisthotics is missing in both specimens, it is not clear if they were in contact with the basioccipital via the processus interfenestralis. The basioccipital forms a flat dorsal surface anteriorly that floors the posterior portion of cavum cranii and contacts the parabasisphenoid. The basioccipital of trionychids is usually underlain by posteriorly directed lappets formed by the parabasisphenoid and the pterygoid. These are not preserved in NDGS 10019, but articular scars left by the pterygoids are visible in NDGS 10029. The width of the basioccipital decreases posterior to its contact with the pterygoids (Fig. 3B, D). The basioccipital tubercles are located halfway along the posterior margin of the skull towards the occipital condyle, which is located dorsally. The basioccipital tubercles of *Gilmoremys lancensis* and *Plastomenus thomasii*, by contrast, are widely placed at the basioccipital-pterygoid contact. A large fossa is located between the basioccipital tubercles, ventral to the occipital condyle. They curve gently dorsally and anteriorly to meet with the exoccipitals (Fig. 6B). Above the basioccipital tubercles, the basioccipital contributes to two of the three foramina hypoglossi.

*Parabasisphenoid* The parabasisphenoid is a single, midline element, that is relatively well-preserved in NDGS 10019 (Figs. 3B, D, 4B, D, 5A, B), but greatly damaged in NDGS 10029 (not figured). As the pterygoids underly this bone, its dorsal exposure is much greater than its ventral exposure (Fig. 5A, B). The parabasisphenoid floors the cavum cranii and contacts the palatines anteriorly, the pterygoids ventrolaterally, the basioccipital posteriorly, and the prootics laterodorsally. The median sella turcica is located in the anterior part of the basisphenoid. The dorsum sellae is relatively low (Fig. 5A, B). The parabasisphenoid forms the lateral wall of the voluminous canalis caroticus internus. The foramina anterius canalis carotici basisphenoidalis (sensu Rollot et al., 2021), which contain the cerebral artery, are widely spaced and have a large diameter and are completely formed by the parabasisphenoid. This condition contrasts with that found in *Plastomenus thomasii*, where the pterygoid forms the ventrolateral aspects of this foramen. The canalis caroticus lateralis, which holds the mandibular artery, is reduced to a large foramen, of which the parabasisphenoid forms the medial margin. As preserved, there are no traces of trabeculae. A lack of bony debris in the relevant area suggests that their absence is genuine, but given the overall preservation of the best-preserved skull, NDGS 10019, we cannot rule out the possibility that this is a result of taphonomy. We similarly cannot identify any clinoid processes, but this, too, may be the result of taphonomy. Trabeculae are well-developed in *P*. *thomasii*, but clinoid processes are missing in that taxon. The canalis nervi abducentis is located posterior to the dorsum sellae and fully enclosed. This differs from the condition seen in *P*. *thomasii*, where the abducens nerve is dorsally exposed.

### Mandible

*General Comments* Four mandibles are available from the Ash Coulee Quarry that differ considerably in size, of which only NDGS 10034 is figured, because it is the most complete (Fig. 7), but the other mandibles are available as 3D models (see Table S1). None are associated with the two available skulls (NDGS 10019 and NDGS 10029) beyond originating from the same quarry. An additional mandible is known from the Judson Locality (Fig. 8).

NDGS 11788 is the largest available mandible (47.2 mm from the anterior tip of the symphysis to the posterior end of the coronoid), but preserves only fragments of the left dental ramus and coronoid. NDGS 10034 is the second largest and best-preserved mandible (57.8 mm from the anterior tip of the symphysis to the posterior end of the left ramus), with the full triturating surface and the complete left ramus completely intact (Fig. 7). All bones are preserved in place, except for the coronoid, which is displaced. The surangular, prearticular, and angular are missing their anterior tips, while the coronoid seems to be missing its posterior end. The articular and retroarticular processes are preserved. NDGS 10084 is the second smallest mandible (39.6 mm from the anterior tip of the symphysis to the preserved end of the left ramus) preserving the articular surfaces in addition to the left coronoid, angular, and prearticular. However, the two latter bones are greatly damaged. NDGS 10329 is the smallest available mandible (25.2 mm from the anterior tip of the symphysis to the preserved end of the left ramus), consisting only of the dentary. In addition to size, the mandible differs substantially in the relative development of the triturating surfaces, with larger individuals having broader crushing surfaces, mostly due to the development of a more elongate symphysis. Splenials are absent in all mandibles, as in all trionychids (Evers et al., 2022; Gaffney, 1979).

*Dentary* The dentary rami are completely fused into a single element, as in most turtles (Gaffney, 1979; Figs. 7, 8). The dentary is the main component of the mandible. It contacts the coronoid dorsally, the surangular posterolaterally, and the prearticular and angular posteromedially. The triturating surfaces are notably flat, though slightly convex (Fig. 7). This differs from *Plastomenus thomasii*, which possesses a dorsal symphyseal mount and distinct labial ridges, and *Gilmoremys lancensis* and *P*. *thomasii*, which exhibit a central depression. The outline of the dentary and the shape of the triturating surfaces changes with size. In the smaller individual (NDGS 10084 and NDGS 10329), the dentary is pointed, the symphysis short, and the posterior triturating surfaces narrow. In the larger specimens (NDGS 10034, Fig. 7, and NDGS 11788), the dentary is rounded anteriorly, the symphysis expanded, and the posterior triturating surfaces expanded. The symphyseal area is relatively long compared with other trionychids, but resembles *G*. *lancensis* and *P*. *thomasii* by nearly reaching the level of the coronoid. In contrast to *G*. *lancensis* and *P*. *thomasii*, however, the dentary lacks a spatulate anterior extension. This extension, however, may have been absent in the larger specimens attributed to *G*. *lancensis* by Joyce et al., (2016). As in all trionychids, the margin of the triturating surface is decorated by numerous nutritive foramina.

The dentary becomes much taller but mediolaterally constricted posterior to the triturating surfaces (Fig. 7). The adductor fossa on the side of the dentary is slightly better developed than in *P*. *thomasii*, mostly because of the posterolateral extension of the triturating surface just anterior to it (Evers et al., 2023). The foramen dentofaciale majus is present at the anterior margin of the fossa, just below the coronoid (Fig. 7C). This foramen remains small, about the size of the nutritive foramina on the ventral side of the dentary. This is unlike *G*. *lancensis* and *P*. *thomasii* in which the foramen dentofaciale majus is proportionally much larger. The dentary thins posteriorly and is reduced to a vertical sheet of bone where it meets the surangular. From this contact it slopes down posteroventrally until a three-way contact with the surangular and angular. The dentary forms almost the entirety of the ventral margin of the mandible, almost reaching the base of the retroarticular process (Fig. 7B).

In medial view (Fig. 7D), the foramen alveolare inferius is located ventral to the anterior end of the coronoid. A deep sulcus is continuous with the foramen posterior to it. Although the anterior part of the prearticular and angular are missing in all specimens, it is likely that the foramen was medially exposed, as it is in extant trionychids (Evers et al., 2022). The sulcus Meckelii is best observed in NDGS 11788 and NDGS 10084. As in extant trionychids and *P*. *thomasii*, the sulcus extends from the fossa Meckelii anteriorly, thins anteriorly, and meets its counterpart medially in the symphyseal area, forming a shallow but distinctly wide notch.

As in all trionychids, the dentary forms an ascending process that covers the anterior margin of the coronoid.

*Coronoid* The coronoid is preserved in NDGS 10034 (Fig. 7), NDGS 11788, and NDGS 10084, though only the left one.

The coronoid is a dorsoventrally high, roughly triangular-shaped bone that forms the distinct coronoid process (Evers et al., 2022; Gaffney, 1979; Meylan, 1987). The coronoid contacts the dentary anteriorly and ventrally and the angular and prearticular posteriorly. The coronoid likely contacted the surangular posterolaterally, but this contact is lost in all specimens. The coronoid is longer than wide (Fig. 7). Three processes can be identified: an anterior process projecting into the dentary, a dorsoposterior process forming the coronoid process, and a posterior process contacting the surangular. A weak but distinct depression is visible between the two posterior processes, but no clear adductor fossa ridge is defined as in *Plastomenus thomasii*. No coronoid foramen is found in any of the specimens. This foramen, present in extant cyclanorbines, is absent in trionychines and *P*. *thomasii* (Evers et al., 2022, 2023). The anterior contact of the coronoid with the dentary cannot be properly observed in any of the specimens examined as this contact is not preserved in any of the available specimens. In NDGS 11788, the coronoid appears to make a minor contribution to the posterior part of the triturating surface, as is also seen in *P*. *thomasii,* but not in *Gilmoremys lancensis*.

*Surangular* The surangular is only preserved in NDGS 10034, but partially damaged (Fig. 7). The surangular participates in the lateral side of the mandible and contacts the dentary ventrally, the angular posteriorly, and the articular medially (Fig. 7). The anterior contact of the surangular with the coronoid is not preserved in this specimen, but was undoubtedly present considering the close proximity of the two bones and the relatively conservative anatomy of the Ash Coulee Quarry mandibles compared with that of other trionychids (Evers et al., 2022). Anteriorly, the surangular is a thin, sheet-like bone forming the lateral margin of fossa Meckelii. The preserved part of fossa Meckelii suggest that it is a narrow anteroposterior opening, as is typically found in trionychids (Evers et al., 2022). At the posterior edge of the fossa, the surangular medially contacts the articular for all its length. At the level of this contact, the surangular forms an ectocondylar flange that contributes to more than half of the mandibular articulation (Fig. 7B). This extended participation of the surangular to the mandibular articulation is a character unique to trionychids (Evers et al., 2022; Meylan, 1987). The dorsal surface of the ectocondylar flange is depressed and dorsolaterally oriented. Posterior to the articulation area, the surangular forms the lateral third of the retroarticular process. The process is weakly depressed and anterodorsally elongated. Alongside the surangular, the angular, articular, and prearticular participate in this process. Unlike the retroarticular process of *Plastomenus thomasii*, the process of *Hutchemys rememdium* is not anteroposteriorly longer than the articular surface. The foramen auriculotemporalis is present ventral to the ectoarticular flange. As in *P*. *thomasii*, it is the posterior end of a canal anteriorly ending in the fossa Meckelii. Other foramina are present at the level of the retroarticular process, as in *P*. *thomasii*, but they do not seem to be directly linked to the foramen auriculotemporalis through a canal.

*Angular* The left angular is preserved in NDGS 10034 (Fig. 7) and NDGS 10084. In the latter specimen, it is heavily fragmented and barely identifiable. NDGS 10034 preserves those portions of the angular that are located anterior to the foramen of fossa Meckelii (Fig. 7).

The angular is a medial, anteroposteriorly elongated bone. The angular contacts the prearticular dorsomedially, the articular dorsally, the surangular anterolaterally, and the dentary ventrally. Anteriorly, the angular is a thin, dorsoventrally high bone. Posteriorly, at the level of the retroarticular process, the angular widens and takes over the dentary to form the ventral margin of the mandible. There the angular underlies the articular in a cup-like structure as it forms the retroarticular process alongside the articular and surangular (Fig. 7D).

*Prearticular* The left prearticular is preserved in NDGS 10034 (Fig. 7), with the anterior part missing, and in NDGS 10084, but greatly fractured.

The prearticular is a medial bone of the mandible. It contacts the articular laterally and the angular ventrolaterally. It is a thin, sheet-like bone forming the lateral margin of fossa Meckelii (Fig. 7). The prearticular curves slightly laterally just posterior to the posterior margin of the fossa Meckelii. As the anterior part of the prearticular is missing, it is unclear if the prearticular and surangular were in contact anteriorly to subdivide the fossa Meckelii, as is observed in some extant trionychids (Evers et al., 2022; Gaffney, 1979; Meylan, 1987). However, according to Meylan, 1987, this is a highly intraspecific variable feature. Usually, the prearticular contacts the coronoid in trionychids (Evers et al., 2022). In NDGS 10034, this part of the mandible is not preserved (Fig. 7), however, it seems that this contact is present in NDGS 10084. The bones are nonetheless too damaged to provide details about this contact. The prearticular borders the mandibular articular surface medially without directly contributing to it and extends shortly into the retroarticular process.

*Articular* The left articular is intact in NDGS 10034 and is overall well-preserved (Fig. 7). It is an anteroposteriorly elongated and dorsoventrally high bone that contacts the surangular laterally, the prearticular medially, and the angular ventrally. Anteriorly, the articular forms the posterior margin of the fossa Meckelii. Ventral to this margin, within the fossa Meckelii, a thin, sheet-like process of the articular projects anteriorly and contacts the surangular. It forms the articular facet of the mandible that connects with the condyle of the quadrate. Lateral to the articular facet, the surangular forms the ectocondylar flange (Figs. 7A, B). The portion of the articular forming the articular facet is less wide than the portion of the surangular. The articular facet is narrow and flatter than that of extant trionychids. The facet contrasts with that of *Plastomenus thomasii*, where it is more convex (Evers et al., 2022, 2023).

Together with the surangular, prearticular, and angular, the articular forms the retroarticular process (Fig. 7). The transition from the articular facet to the retroarticular process is marked by a faint ridge and a difference in bone texture from a spongy to smooth texture. This resembles the condition described by Evers et al. (2022) for extant trionychids. The retroarticular process is anteroposteriorly elongated, as in all trionychids (Gaffney, 1979; Meylan, 1987). Anteriorly, at the margin of the mandibular articulation, the retroarticular process is slightly convex and then it becomes slightly concave posteromedially. Much like the articular facet, the retroarticular process of *Hutchemys rememdium* is flat compared to modern relatives and does not show the distinct articular notch found in extant species (Evers et al., 2022).

### Shell

*General Comments* The shell of *Hutchemys rememdium* was previously described by Joyce et al. (2009) and Hutchison (2009), as his *Plastomenoides lamberti*, based on the type specimen from the Torrejonian of Montana. In the section below, we first summarize the morphology of the new shell material from North Dakota and make explicit comparisons with the type material in the discussion. The available shells range in size from approximately 21 cm (NDGS 11879) to approximately 40.5 cm (SMM P97.8.26).

*Carapace* As in most plastomenids, the trionychid shells from North Dakota are composed of a single unpaired nuchal, eight pairs of costals, a preneural, and seven to eight neurals (Figs. 9, 10, 11A, B, 12A). The carapace outline is oval, longer than wide, with a deep nuchal notch, and a flat posterior margin. The surface ornamentation consists of interlocking ripples that follow the outline of the carapace. The ornamentation gradually fades toward the center of the carapace, which is nearly smooth.

The nuchal is the anteriormost element of the carapace (Figs. 9, 10C, 11A, B). The costiform processes, which are the deep portion of the nuchal, are fully covered in all specimens by surficial metaplastic bone. The costiform processes expand laterally into wing-like processes, but there is no evidence of them forming a lateral comb consisting of many fingerlike projections. The paired ventral depressions that provide space for the articulation of the cervical vertebrae with the thoracic vertebral column are located in the middle of the anterior half of the nuchal. The metaplastic ossification portion of the nuchal is deeply inset within the costal series. The anterior margin is thinner than the rest of the bone and slightly overhangs costal I laterally to form a distinct notch along the anterior margin of the carapace. In NDGS 1203 (Fig. 9A) and NDGS 10028 (Fig. 9B), the nuchal callosity is four times wider than long, a plastomenid apomorphy. However, in smaller specimens, such as NDGS 10625 (Fig. 9C) and NDGS 11879 (Fig. 9D), the nuchal is proportionally longer and thinner, only about three times wider than long.

The costals are ossified for almost the full length of the underlying endochondrally ossified ribs (Figs. 9, 10, 10B, 11A, B, 12A). Costal I is large and curves anteriorly, following the shape of the nuchal and contacting it fully. No suprascapular fontanelles are present in any of the available specimens. Costal II is also anteriorly curved, but to a much lesser degree than costal I. Costal II widens toward the lateral edge of the carapace in all specimens. Costal III is slightly curved toward the anterior of the carapace while costal IV is straight. Costals V, VI, and VII are curved posteriorly. Costal VIII is a triangular element, but much variation is apparent in regards to its shape and size. In NDGS 1203 (Fig. 9A), NDGS 10028 (Fig. 9B), NDGS 12215 (Fig. 10A), and the left side of NDGS 11879 (Fig. 9D) and SMM P91.17.1 (Fig. 12A), costal VIII is wider than long. In SMM P97.8.26 (Fig. 11A), costal VIII is about as long as wide. Finally, in NDGS 10057 (Fig. 10B), NDGS 10115 (Fig. 10C), NDGS 10625 (Fig. 9C), and the right sides of NDGS 11879 (Fig. 9D) and SMM P91.17.1 (Fig. 12A), costal VIII is longer than wide. No correlation is apparent between the size of the specimen and the relative shape of costal VIII. Costals I–VI are fully separated from their counterparts by the neural series. The midline contact of costals VII varies depending on the development of neural 7 and/or the presence of neural 8 (see below). In NDGS 1203 (Fig. 9A), NDGS 10028 (Fig. 9B), NDGS 10057 (Fig. 10B), NDGS 10625 (Fig. 9C), NDGS 11879 (Fig. 9D), NDGS 12215 (Fig. 10A), and NDGS 11790 (Fig. 10D) costals VII partially contact one another posterior to neural 7. In SMM P91.17.1 (Fig. 12A) a regular neural 7 and a small neural 8 hinder a midline contact of costals VII. Finally, in SMM P97.8.26 (Fig. 11A) and NDGS 10115 (Fig. 10C) an enlarged neural 7 fully subdivides costals VII. The complicated, polygonal outline of this element suggests that this is perhaps a fused neural 7 and 8, but this cannot be confirmed, as all connections with the underlying vertebrae are lost. Costals VIII always contact each other medially along their full length. The posterior margin of the carapace is either rounded to flat (e.g., NDGS 10625, Fig. 9C) or presents a broad, but shallow median concavity within costal VIII (e.g., NDGS 1203, Fig. 9A; and NDGS 10028, Fig. 9B). Within the population from the Ash Coulee quarry, these notches appear to be mostly present in large specimens, but the notch is absent in the even larger specimen found near Wannagan (SMM P97.8.26; Fig. 11A). All specimens exhibit thickened, grooved costal margins (i.e., “split costals” of Joyce et al., 2009). In NDGS 1203 (Fig. 9A) and NDGS 10028 (Fig. 9B), the splitting is even from the front of the shell to the back, as in the holotype of *Hutchemys rememdium* (Joyce et al., 2009). In NDGS 10625 (Fig. 9C), NDGS 11879 (Fig. 9D), and SMM P97.8.26 (Fig. 11B) splitting is distinctly asymmetric towards the back of the shell, in particular round costal VI, in that the surficial aspect of the bone far outgrows the visceral side. This resembles the type material of *Hutchemys arctochelys* (Joyce et al., 2009). There is no apparent correlation between the symmetry of costal splitting and the relative size of the carapace.

The neural series is typically comprised of one preneural and seven neurals (NDGS 1203, Fig. 9A; NDGS 10028, Fig. 9B; NDGS 10057, Fig. 10B; NDGS 10625, Fig. 9C; NDGS 11879, Fig. 9D; NDGS 12215, Fig. 10A; NDGS 11790, Fig. 10D). While a neural 8 is clearly developed in one individual (SMM P91.17.1, Fig. 12A), a prolonged neural 7 is found in two others, that resembles a fused neural 7 + 8 (NDGS 10115, Fig. 10C; SMM P97.8.26, Fig. 11A). In these last two morphotypes, a midline contact of costals VII is prevented by the neural series. In all individuals, the preneural is trapezoidal and wider than long, neural 1 is a small, rectangular element, neural 2 is a large, octagonal element, neurals 3–5 are hexagonal with short posterior sides, the reversal occurs at neural 6, and neural 7 is pentagonal with short anterior sides.

*Plastron* The plastron of the trionychid shells from North Dakota forms a fully ossified shield devoid of fontanelles. In ventral view, the callosities fully cover the deep portions of the plastral bones and, except for the epiplastra, all elements of the plastron are fully sutured to one another (Figs. 8B, D, 11C, D, E, 12B, C, 13, 14). The ornamentation on the plastron, much like that of the carapace, fades from the bridges toward the center. The paired epiplastron is the only element not enclosed by another. A large sample of isolated epiplastra from the Ash Coulee site (Fig. 14G, H, I, J, K, L, M, N) provides much information about this element. In contrast to *Plastomenus thomasii* (Hutchison, 2009) and *Plastomenus joycei* (Lyson et al., 2021), the epiplastral processes are not I-shaped, but rather J-shaped, as in all trionychines and sometime observed in cyclanorbines. The posterior epiplastral process is much shorter than the anterior process, which combined gives the processes the outline of a hockey-stick or small j, best seen in NDGS 9947, (Fig. 14G), NDGS 11681 (Fig. 14J), NDGS 11685 (Fig. 14M), NDGS 12226 (Fig. 14N). The epiplastral callosities range from longer than wide (e.g., NDGS 11681, Fig. 14J) to wider than long (e.g., NDGS 12226, Fig. 14N). As the anterior process of most elements is similar, we find no correlation between the size of the callosity and the size of the element. The entoplastral callosity is rectangular in ventral view and wider than long (Figs. 11C, D, 12B, 13, 14A, B, C, D, E, F). It is posteriorly and laterally enclosed completely by the hyoplastron and forms the straight to slightly convex anterior margin of the plastron. In dorsal view, the boomerang shaped, deep bony tissue of the entoplastron is easily distinguished from the surficial metaplastic callosity (e.g., Figs. 13B, E, 14A, D). The posterolateral ends of the entoplastral processes are ventrally covered by the hypoplastral callosity upon which they leave an imprint (e.g., Figs. 11D, 12C, 13A, C, D, F, 14C, F). The hyoplastra fully contact each other medially, fully suture (but do not fuse) with the hypoplastron posteriorly, and fully surround the posterior and lateral margins of the entoplastron. The hyoplastron has a single lateral process, which, when intact, always projects minorly beyond the margin of the callosity (Figs. 12C, 14A, C, D, F). The medial processes cannot be traced in any individual. The hyo-hypoplastral callosities jointly form the straight to slightly convex lateral margin of the plastron. There is no evidence of surficial sculpturing on the dorsal side of this margin. The hyoplastral shoulder is well-developed and connects the lateral process to the anterior margin of the plastron. The shoulder is slightly concave or straight and does not extend further anteriorly than the entoplastron. The hypoplastra are sutured to each other medially, the hyoplastra anteriorly, and the xiphiplastra posteriorly. The hypoplastron has two lateral processes that universally protrude beyond the margin of hypoplastral callosity. The medial processes cannot be traced in any specimen. While in ventral view, the hypo-xiphiplastral contact is a straight horizontal contact, in visceral view, the posterior process of the hypoplastron accommodates two anterior processes of the xiphiplastron. The xiphiplastron fully contacts its counterpart medially. The xiphiplastron forms the rounded lobe as the posterior margin of the plastron. The xiphiplastron varies from wider than long to about as wide as long. The xiphiplastral processes can be distinguished from the underlying callosity in some specimens, in particular SMM P77.6.99 (Fig. 8D) and NDGS 18196 (Fig. 13C, F). These specimens highlight the presence of two anterior processes, which clasp the large posterior process of the hypoplastron, a single, posterior process that curves posteromedially to nearly contacts its counterpart along the midline, and a short, medial process that does not contact its counterpart (Fig. 12C). A rounded band of metaplastic bone protrudes beyond the level of the xiphiplastral processes to form the broadly rounded posterior margin of the plastron.

### Axial and appendicular skeleton

In addition to cranial elements, a selection of limb bones and vertebrae were collected from the Ash Coulee Quarry and µCT scanned to produce 3D models (Fig. 16). Additional, redundant elements are available at NDGS, though typically show more damage.

NDGS 10441 (Fig. 16C) is a partial eighth cervical vertebra. It is similar to that of other trionychids by being wide and flat. The postzygapophyses are widely spaced. The centrum is strongly flattened. Its ventral side is flat and lacks a median spine. A posterior articulation with the first thoracic vertebra is not developed. The neural arch is faintly rounded and shows no neural spine.

NDGS 18193 (Fig. 16D) and NDGS 10435 (Fig. 16E) are caudal vertebrae. The centrum is long and hourglass-shaped in ventral view. The opisthocoelous articular surfaces are twice as wide as tall. The anterior facet is strongly concave while the posterior one is strongly convex. NDGS 18193 has tightly spaced postzygapophyses and well-developed transverse processes suggesting an origin from the anterior portion of the caudal vertebral column. The prezygapophyses are damaged. NDGS 10435 has larger, wider spaced postzygapophyses, but smaller, though damaged transverse processes more consistent with an origin from the posterior portion of the caudal vertebral column. The prezygapophyses extend anteriorly well past the centrum. The neural spine is low and rugose in both specimens.

NDGS 18191 is a right pectoral girdle (Fig. 16A) only lacking the medial end of the coracoid. The scapula and acromion are tightly sutured to one another. While the scapular process and glenoid are strap-like, the coracoid is expanded into a rounded blade.

NDGS 10092 (Fig. 16B) is a left pelvic girdle. The medial and pectineal processes are damaged and the original shape is distorted by minor crushing. The single thyroid fenestra was likely ovoid and wider than long. The pectineal process of the pubis is large and curves anteromedially, the common condition seen in most trionychids. It is unclear if the pectineal process reached further anteriorly than the medial process. The ilium forms a robust dorsal process that projects dorsally and bends posteriorly at mid length. We cannot ascertain the presence or development of a metischial process as the ischium is badly damaged.

The femur and humerus of trionychids resemble one another closely but can be differentiated by the presence of the ectepicondylar foramen on the distal part of the humerus. The medial process of the humerus is larger than the trochanter major. This is observed when comparing right humerus NDGS 11531 (Fig. 16F) to left femora NDGS 10361 (Fig. 16H), NDGS 10559 (Fig. 16G), and NDGS 10008 (Fig. 16I). The shaft of both humerus and femur are strongly S-shaped. The available tibiae (NDGS 10338, Fig. 16J; NDGS 10560, Fig. 16K; NDGS 10511, Fig. 16L) and left radius (NDGS 18192, Fig. 16O) are rod-like elements with slightly expanded ends. The fibulae (NDGS 10613, Fig. 16M; NDGS 10157, Fig. 16N) are straight and rod-like. The available claw (NDGS 11481, Fig. 16P) resembles that of other trionychids by being relatively broad and flat.

## Results of phylogenetic analysis

**Fig. 17:**
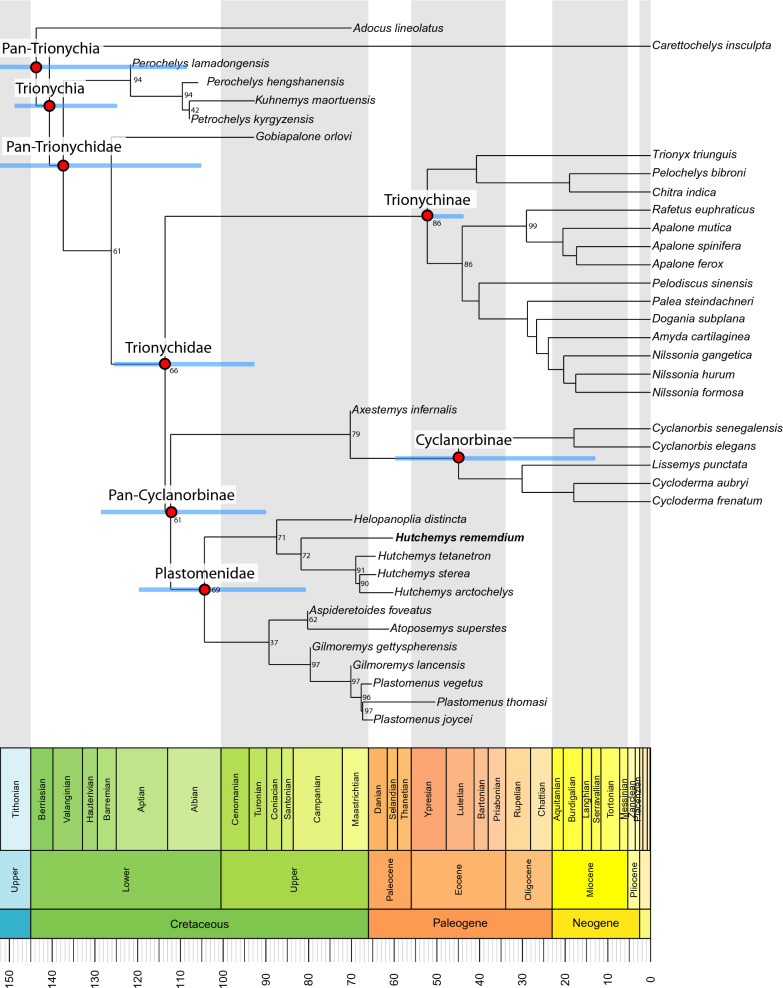
Phylogenetic topology of the Monte Carlo Chain MCMC tree retrieved from the Bayesian tip-dating analysis. Terminal end of branch for each taxon is scaled to geological time, major clades labeled and shown with a 95% highest posterior density interval for the estimated node age, numbers show clade credibility value, and unlabeled nodes indicate clade credibility is 100%

*Bayesian analysis* The MCMC tree resulting from the Bayesian analysis is a half compatibility tree, which collapses nodes under 50% credibility (Fig. 17). In this phylogram, the relationships of all clades are completely resolved, but some nodes show low support.

*Plastomenidae* is recovered as the most basal clade within *Pan-Cyclanorbinae*. This relationship, however, is weakly supported, with a node credibility at 56%, which means this node is only recovered in just over half of the trees. Within *Plastomenidae*, *Hutchemys* spp*.* + *Helopanoplia distincta* form a clade that is sister to *Atoposemys superstes* + *Aspideretoides foveatus* + *Gilmoremys* spp. + *Plastomenus* spp. *Axestemys infernalis* is recovered as the immediate sister of *Cyclanorbinae*. All Early Cretaceous pan-trionychids are found along the stem of *Trionychidae*. Relationships are fully resolved within the *Helopanoplia* + *Hutchemys* clade, but do not replicate the stratigraphic appearance of various taxa, which is notable as the tree otherwise follows stratigraphy closely. See the complete MrBayes log in Additional file 6.

**Fig. 18:**
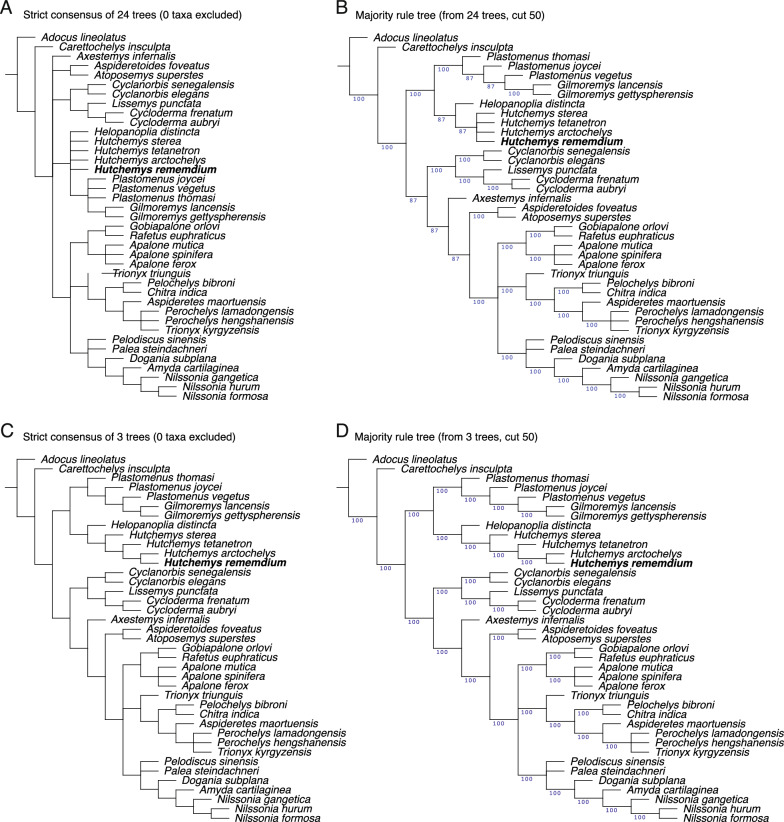
Phylogenetic results from parsimony analysis. **A**, Strict consensus tree of unweighted analysis; **B**, majority rule tree of unweighted analysis; **C**, strict consensus tree of weighted K = 12 analysis; **D**, majority rule tree of weighted K = 12 analysis

*Parsimony analysis* The equal weighting analysis recovered 24 MPTs with 398 steps (CI = 0.362; RI = 0.582; Fig. 18A). In the strict consensus tree, *Plastomenidae* is recovered in a polytomy with *Cyclanorbinae* and *Trionychinae*. *Hutchemys* spp. Are recovered in an unresolved clade with *Helopanoplia distincta* and *Plastomenus* spp. + *Gilmoremys* spp. Clade. *Atoposemys superstes* + *Aspideretoides foveatus* and *Axestemys infernalis* are recovered outside *Cyclanorbinae*, *Plastomenidae*, and *Trionychinae*. Various Early Cretaceous pan-trionychids are recovered deep within *Trionychinae*.

In the Majority Rule topology (Fig. 18B), *Plastomenus* spp.,* Gilmoremys* spp*.*, *Hutchemys* spp., and *Helopanoplia distincta* are recovered as plastomenids along the stem of *Trionychidae*. This clade contains two distinct lineages, the *Gilmoremys*/*Plastomenus* lineage and the *Helopanoplia*/*Hutchemys* lineage. *Axestemys infernalis* and *Atoposemys superstes* + *Aspideretoides foveatus* are recovered as the most basal pan-trionychines*.* Various Early Cretaceous pan-trionychids are recovered in the same position as in the strict consensus.

The weighted analysis (K = 12) resulted in 3 MPTs with an implied length of 398 steps (best score 16.19; CI = 0.362; RI = 0.582). The strict consensus and majority rule topologies are the same (Fig. 18C, D). The weighted topologies are similar to the equal-weight majority rule topology. The only difference is that the down weighted topology has more resolved relationships. Notably, the interrelationships of the *Helopanoplia* + *Hutchemys* clade fully mirror the stratigraphic appearance of these taxa from the late Maastrichtian to the late Paleocene.

Exploratory runs using TNTs “traditional search” option yielded the same number of trees. Similarly, searches with K-values of 9, 6, and 3 yielded similar results as well. See the complete set of trees in Additional files 3, 4, 5.

## Discussion

### Alpha taxonomy

All available plastomenid material described herein from North Dakota can readily be referred to *Hutchemys* as all carapaces possess an octagonal neural 2 and because the entoplastral callosity is fully embedded laterally between the hyoplastra (Hutchison, 2009; Joyce et al., 2009; Vitek & Joyce, 2015). At present, only two species are known from the middle to late Paleocene: *Hutchemys rememdium*, which is based on Torrejonian to Tiffanian 3 material, and *Hutchemys arctochelys*, which is based on Clarkforkian 1 material (Joyce et al., 2009). As originally conceived, *Hutchemys rememdium* is the smaller of the two species, has a deeper nuchal notch, evenly split costals, a smaller neural 1, and costals VIII that are wider than long, while *Hutchemys arctochelys* is much larger, has a less distinct nuchal notch, asymmetrically split posterior costals, a constriction at the level of costal V, a larger neural 1, costals VIII that are far longer than wide, surficial sculpturing on the dorsal side of the plastron and on the ventral side of the carapace, and distinct bony flaps behind the inguinal processes of the hypoplastron.

The new material from North Dakota all originates from the Tiffanian 4 and somewhat bridges the known morphology of *Hutchemys rememdium* and *Hutchemys arctochelys* in that the depth of the nuchal notch varies, the costals are either evenly split or asymmetric, neural 1 is well-developed, costals VIII range from wider than long to longer than wide, but there is neither evidence of a constriction, nor unusual sculpturing, or bony flaps on the hypoplastron. The largest specimens are intermediate in size. Although it may seem plausible to name a new, intermediate Tiffanian chronospecies that links the Torrejonian *Hutchemys rememdium* with the Clarkforkian *Hutchemys arctochelys*, we note that the large, new population of Tiffanian 4 material from North Dakota can easily encompass the Torrejonian type of *Hutchemys rememdium*, while remaining easily distinguishable from all Clarkforkian material of *Hutchemys arctochelys* by being notably smaller and lacking any signs of hyperossification, including particularly elongate costals VIII, surficial sculpturing on the dorsal side of the plastron and on the ventral side of the carapace, and distinct bony flaps behind the inguinal processes of the hypoplastron. We, therefore, here refer the new material from North Dakota to *Hutchemys rememdium* and slightly rephrase the diagnoses of both species (see Systematic Paleontology above). As all apparent morphotypes form a trend through time with an increase in size and ossification, it is plausible that late Paleocene *Hutchemys* in the greater Williston basin represent a single anagenetic lineage, which we here split into two chronospecies.

### Circulatory pattern in the snout

**Fig. 19:**
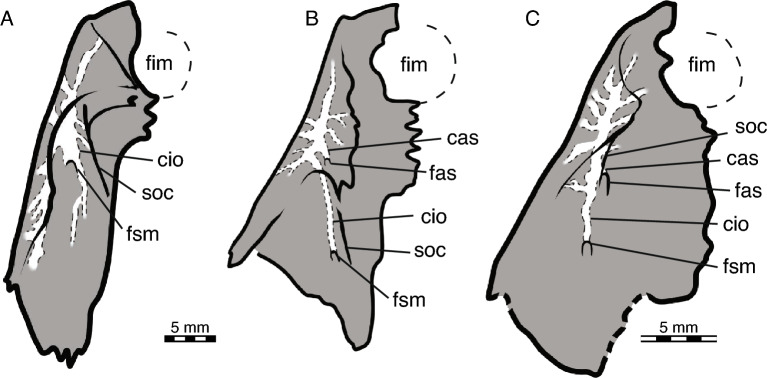
Illustrations of the circulatory system in the maxillae of trionychids. **A**, The cyclanorbine *Lissemys ceylonensis*, NMB 2398; **B**, the trionychine *Dogania subplana*, PCHP 2919; **C**, the plastomenid *Hutchemys rememdium*, NDGS 10019. *cio* canalis infraorbitalis, *cas* canalis alveolaris superior, *fas* foramen alveolare superius, *fim* foramen intermaxillare, *fsm* foramen supramaxillare, *soc* suborbital crest

Canals present in the bones of the skull of *Hutchemys rememdium* allows us to infer the circulatory pattern of this taxon and to compare it with that other trionychids. In trionychines, two medial foramina supply the canalis infraorbitalis: the foramen supramaxillare within the orbit and the more anterior foramen alveolare superius, which often enters within the nasal cavity (Albrecht, 1967; Fig. 19B). In cyclanorbines, on the other hand, the foramen alveolare superius is absent (Evers et al., 2023; Fig. 19A).

In their description of *Plastomenus thomasii*, Evers et al. (2023) identify a fenestra within the nasal cavity (= supraalveolare fenestra) that they consider to be non-homologous to the foramen alveolare superius, as its shape is different and no clear sulcus that would indicate the entry of an artery. As this morphology correlates with the development of a more expressed suborbital crest, they furthermore suggest that the infraorbital artery is hindered from entering the nasal cavity in this taxon by this ridge. *Plastomenus thomasii*, therefore, appears to share the absence of the infraorbital artery with cyclanorbines. The phylogenetic analyses of Evers et al. (2023) place *Hutchemys* spp. within *Pan-Cyclanorbinae* bracketed by *P*. *thomasii* and *Cyclanorbinae*. *Hutchemys* spp. are therefore predicted to lack an infraorbital artery as well.

The new skull material of *Hutchemys rememdium* described herein unambiguously shows that this turtle exhibits the trionychine pattern regarding the maxillary blood supply (Figs. 6C, 19C). In some trionychines, in addition, no obvious sulci precede the foramina supramaxillare and alveolare superius (*Chitra chitra* NHMUK 1936.12.16.1; *Pelochelys cantorii* NMB 11985, PCHP 4974) and we also find a fenestra-like shape of the foramen alveolare superius in *Chitra chitra* (NHMUK 1936.12.16.1), with no suborbital crest. Lastly, several species of trionychines show a well-developed suborbital crest that should hinder the development of an infraorbital artery (*Amyda cartilaginea* FMNH244117; *Apalone spinifera* FMNH 22178; *Palea steindachneri* MTD 33802; *Pelodiscus sinensis* NMB 1438). Indeed, the suborbital crest of some trionychines is even more distinct than that in *P*. *thomasii* (*Palea steindachneri* MTD 33802; *Pelodiscus sinensis* NMB 1438). It, therefore, appears clear that not all plastomenids have a pattern reminiscent of that of cyclanorbines and that there is no correlation between the development of a suborbital crest and the loss of an infraorbital artery. In the specific case of *P. thomasii*, the very presence of an opening to the canalis infraorbitalis suggests the entry of a blood vessel, even if a clear path cannot be discerned. Given the great amounts of variation found in the skull of trionychids, we suspect that additional specimens of this taxon may show a pattern more reminiscent of trionychines. We, therefore, await description of additional plastomenid crania while remaining cautious about the evolutionary significance of this anatomical system.

### Geniculate ganglion

Rollot et al. (2021) identified two patterns for the split of the facial nerve among trionychids. In the first case, the geniculate ganglion is located outside of the prootic within the canalis cavernosus and the vidian nerve passes the pterygoid through the canalis pro ramo nervi vidiani to enter the internal carotid (see Rollot et al., 2021, Fig. 18, facial pattern IA and IIA). This pattern was found in their sample of amydines (i.e., *Amyda cartilaginea*, *Pelodiscus sinensis*) and apalonines (i.e., *Apalone spinifera* [include their *Apalone mutica*, which actually represents *Apalone spinifera* as well]). In the second pattern, the ganglion is located within the prootic, between the canalis cavernosus and the canalis caroticus internus. This was seen in the available sample of chitrines (i.e., *Chitra chitra*) and cyclanorbines (i.e., *Cyclanorbis senegalensis*, *Cycloderma frenatum*, and *Lissemys punctata*). In *Hutchemys rememdium*, the ganglion is located in the prootic.

Ignoring the observation that this pattern is also found in *Chitra indica*, the position of the geniculate ganglion seems to indicate a phylogenetic split between trionychines and cyclanorbines and suggests close relationships of *H*. *rememdium* with cyclanorbines. However, in the closely related *P*. *thomasii*, the geniculate ganglion is present within the canalis cavernosus, suggesting relationships with trionychines, if this character is taken at face value. This highlights, once again, much variability within closely related groups. A study of the evolutionary history of this character using a much broader sample should be of interest.

### Phylogeny and evolution of *Pan-Trionychidae*

The taxon *Plastomenidae* was originally coined by Hay (1902, 1908) to group an assortment of Late Cretaceous to Eocene trionychids from North America that exhibit a plastron with broad callosities that connect to form a solid shield. Over the course of the last decade, a number of phylogenetic hypotheses focusing on the best-preserved taxa typically confirmed this grouping to be monophyletic (e.g., Edgar et al., 2022; Evers et al., 2023; Jasinski et al., 2022; Joyce & Lyson, 2017; Joyce et al., 2016, 2018; Lyson et al., 2021; Vitek et al., 2018). However, there has been little agreement as to the internal relationships of this clade. Although the recently described skulls of *Gilmoremys lancensis* and *Plastomenus thomasii* might have been expected to provide meaningful insights, in particular as the two are so similar, cranial morphology apparently has thus far had no impact on plastomenid phylogeny, beyond confirming the monophyly of the group relative to other turtles with preserved cranial material.

We here describe the first known skulls for any representative of the *Hutchemys* clade, referable to *Hutchemys rememdium*. Despite all apparent differences in overall gestalt, the skull of *Hutchemys rememdium* resembles those of *Gilmoremys lancensis* and *Plastomenus thomasii* by having a secondary palate, a feature not seen to that degree in any other group of trionychids. Nonetheless, while the skulls of *Gilmoremys lancensis* and *Plastomenus thomasii* exhibit fused frontals and reduced to absent postorbitals, the frontals of *Hutchemys rememdium* are unfused and the postorbitals are notably large. So, while *Gilmoremys lancensis* and *Plastomenus thomasii* were sometimes, but not always, placed as each other’s immediate sisters in previous trees (Edgar et al., 2022; Evers et al., 2023; Jasinski et al., 2022; Joyce & Lyson, 2017; Joyce et al., 2016, 2018; Lyson et al., 2021; Vitek et al., 2018), we consistently find this result in our analyses. In other words, the inclusion of the skull of *Hutchemys rememdium* enforces the idea that *Gilmoremys* spp. and *Plastomenus* spp. form a monophyletic lineage that ranges from the Campanian to Eocene. This hypothesis is further supported by the observation that *Gilmoremys* spp. and *Plastomenus* spp. possess two hyoplastral processes, in additional to carapacial striations, while other all plastomenids possess only a single hyoplastral process and lack carapacial striations.

Interestingly, although plastomenids were initially named to unite trionychids with well ossified plastra, many representatives of the group only possess relatively poorly ossified plastra, including *Aspideretoides foveatus*, *Atoposemys superstes*, and *Gilmoremys* spp. The recognition of a *Gilmoremys*/*Plastomenus* lineage implies that the heavily ossified plastron of *Plastomenus thomasii* was acquired independently from that of *Hutchemys* spp. This is supported by the somewhat different bauplan of the two groups, with *Hutchemys* spp. having an enlarged entoplastral callosity surrounded by the particularly enlarged anterior hyoplastral lobes. This suggests a strong evolutionary pressure towards acquiring more strongly ossified shells for plastomenids in the Late Cretaceous to early Paleogene of North America.

### Palaeoecology

All *Hutchemys rememdium* material described herein was collected from several Tiffanian 4 localities distributed across the western half of North Dakota. In all cases, these localities are interpreted as low energy swamps, lakes, or ponds (see Materials above). Although rich remains of other turtles were recovered from some of these localities, in particular Wannagan Creek, we find notable that the trionychid fauna appears to be dominated by, indeed perhaps only consisting of, *Hutchemys rememdium*. This is striking as trionychid faunas in the underlying (e.g., the Campanian Kaiparowits and Dinosaur Park formations, the Maastrichtian Hell Creek Formation, the Puercan Nacimiento and Denver formations) and overlying formations (e.g., Bridger Formation) are particularly diverse (see Vitek & Joyce, 2015 for a summary). This suggests, at the very least, that *Hutchemys rememdium* favored non-riverine waters and a scarcity of rivers in the Sentinel Butte and Bullion Creek formations, but the homogeneity of the fauna is puzzling nevertheless.

The skull of *Hutchemys rememdium* is notable for being relatively short, but exhibiting broad triturating surfaces. This is consistent with a durophagous diet that includes small mollusks and crustaceans (Dalrymple, 1977; Foth et al., 2017).

## Supplementary Information

**Additional file 1.** Material S1: Table of individual 3D models with associated Links and ARK identifier.

**Additional file 2.**Character-taxon matrix.

**Additional file 3.** TNT script.

**Additional file 4.** Complete set of most parsimonious trees obtained in the equal weighting parsimony analysis.

**Additional file 5.** Complete set of most parsimonious trees obtained in the weighted K=12 parsimony analysis.

**Additional file 6.** txt: MrBayes log.

## Data Availability

All µCT data and 3D models with scanning parameters are available at MorphoSource (https://www.morphosource.org/projects/000594396). Additionally, individual link to each model is provided in Supplementary Material S1.

## Abbreviations

AMNH: American Museum of Natural History, New York, New York, U.S.A
BMRP: Burpee Museum of Natural History, Rockford, Illinois, U.S.A
FMNH: Field Museum of Natural History, Chicago, Illinois, USA
MTD: Museum für Tierkunde Dresden, Dresden, Germany
MVZ: Museum of Vertebrate Zoology, Harvard University, Cambridge, Massachusetts, U.S.A
NDGS: North Dakota State Fossil Collection, Bismarck, North Dakota, U.S.A
NHMUK: Natural History Museum, London, United Kingdom
NHMW: Naturhistorisches Museum Wien, Vienna, Austria
NMB: Naturhistorisches Museum Basel, Basel, Switzerland
PCHP: Peter C.H. Pritchard Collections, Turtle Conservancy, Ojai, California, U.S.A
SMF: Senckenberg Naturmuseum, Frankfurt, Germany
SMM: Science Museum of Minnesota, Saint Paul, Minnesota, U.S.A
YPM: Peabody Museum of Natural History, New Haven, Connecticut, U.S.A
