# Supplementary material for: The cranial and postcranial morphology of Hutchemys rememdium and its impact on the phylogenetic relationships of Plastomenidae (Testudinata, Trionychidae)
Source: Swiss J Palaeontol. 2024 May 24;143(1):22. doi: 10.1186/s13358-024-00315-8 (PMC11126460; doi:10.1186/s13358-024-00315-8)
Supplement: Supplementary file 1 — Additional file 1. Material S1: Table of individual 3D models with associated Links and ARK identifier. [file 13358_2024_315_MOESM1_ESM.docx]

| 3D Models | | | |
| --- | --- | --- | --- |
| Catalog Number | Description | ARK HyperLink | ARK Link |
| NDGS 10001 | Plastron | [ark:/87602/m4/597350](http://n2t.net/ark:/87602/m4/597350) | http://n2t.net/ark:/87602/m4/597350 |
| NDGS 10008 | Left Femur | [ark:/87602/m4/597252](http://n2t.net/ark:/87602/m4/597252) | http://n2t.net/ark:/87602/m4/597252 |
| NDGS 10019 | Basioccipital | [ark:/87602/m4/594399](http://n2t.net/ark:/87602/m4/594399) | http://n2t.net/ark:/87602/m4/594399 |
| NDGS 10019 | Canalis Caroticus Internus | [ark:/87602/m4/595955](http://n2t.net/ark:/87602/m4/595955) | http://n2t.net/ark:/87602/m4/595955 |
| NDGS 10019 | Canalis Cavernosus | [ark:/87602/m4/595958](http://n2t.net/ark:/87602/m4/595958) | http://n2t.net/ark:/87602/m4/595958 |
| NDGS 10019 | Canalis Nervus Abducentis | [ark:/87602/m4/595967](http://n2t.net/ark:/87602/m4/595967) | http://n2t.net/ark:/87602/m4/595967 |
| NDGS 10019 | Canalis Nervus Facialis | [ark:/87602/m4/595961](http://n2t.net/ark:/87602/m4/595961) | http://n2t.net/ark:/87602/m4/595961 |
| NDGS 10019 | Canalis Nervus Vidianus | [ark:/87602/m4/595964](http://n2t.net/ark:/87602/m4/595964) | http://n2t.net/ark:/87602/m4/595964 |
| NDGS 10019 | Cranium | [ark:/87602/m4/594394](http://n2t.net/ark:/87602/m4/594394) | http://n2t.net/ ark:/87602/m4/594394 |
| NDGS 10019 | Left Epipterygoid | [ark:/87602/m4/594402](http://n2t.net/ark:/87602/m4/594402) | http://n2t.net/ark:/87602/m4/594402 |
| NDGS 10019 | Left Exoccipital | [ark:/87602/m4/594405](http://n2t.net/ark:/87602/m4/594405) | http://n2t.net/ark:/87602/m4/594405 |
| NDGS 10019 | Left Frontal | [ark:/87602/m4/594408](http://n2t.net/ark:/87602/m4/594408) | http://n2t.net/ark:/87602/m4/594408 |
| NDGS 10019 | Left Jugal | [ark:/87602/m4/594417](http://n2t.net/ark:/87602/m4/594417) | http://n2t.net/ark:/87602/m4/594417 |
| NDGS 10019 | Left Maxilla | [ark:/87602/m4/594425](http://n2t.net/ark:/87602/m4/594425) | http://n2t.net/ark:/87602/m4/594425 |
| NDGS 10019 | Left Opisthotic | [ark:/87602/m4/594429](http://n2t.net/ark:/87602/m4/594429) | http://n2t.net/ark:/87602/m4/594429 |
| NDGS 10019 | Left Palatine | [ark:/87602/m4/594432](http://n2t.net/ark:/87602/m4/594432) | http://n2t.net/ark:/87602/m4/594432 |
| NDGS 10019 | Left Parietal | [ark:/87602/m4/594435](http://n2t.net/ark:/87602/m4/594435) | http://n2t.net/ark:/87602/m4/594435 |
| NDGS 10019 | Left Postorbital | [ark:/87602/m4/594438](http://n2t.net/ark:/87602/m4/594438) | http://n2t.net/ark:/87602/m4/594438 |
| NDGS 10019 | Left Prefrontal | [ark:/87602/m4/594441](http://n2t.net/ark:/87602/m4/594441) | http://n2t.net/ark:/87602/m4/594441 |
| NDGS 10019 | Left Prootic | [ark:/87602/m4/594445](http://n2t.net/ark:/87602/m4/594445) | http://n2t.net/ark:/87602/m4/594445 |
| NDGS 10019 | Left Pterygoid | [ark:/87602/m4/594448](http://n2t.net/ark:/87602/m4/594448) | http://n2t.net/ark:/87602/m4/594448 |
| NDGS 10019 | Left Quadrate | [ark:/87602/m4/594452](http://n2t.net/ark:/87602/m4/594452) | http://n2t.net/ark:/87602/m4/594452 |
| NDGS 10019 | Left Quadratojugal | [ark:/87602/m4/594455](http://n2t.net/ark:/87602/m4/594455) | http://n2t.net/ ark:/87602/m4/594455 |
| NDGS 10019 | Parabasisphenoid | [ark:/87602/m4/594458](http://n2t.net/ark:/87602/m4/594458) | http://n2t.net/ark:/87602/m4/594458 |
| NDGS 10019 | Premaxilla | [ark:/87602/m4/606342](http://n2t.net/ark:/87602/m4/606342) | http://n2t.net/ark:/87602/m4/606342 |
| NDGS 10019 | Right Epipterygoid | [ark:/87602/m4/606338](http://n2t.net/ark:/87602/m4/606338) | http://n2t.net/ark:/87602/m4/606338 |
| NDGS 10019 | Right Exoccipital | [ark:/87602/m4/606340](http://n2t.net/ark:/87602/m4/606340) | http://n2t.net/ark:/87602/m4/606340 |
| NDGS 10019 | Right Frontal | [ark:/87602/m4/606345](http://n2t.net/ark:/87602/m4/606345) | http://n2t.net/ark:/87602/m4/606345 |
| NDGS 10019 | Right Jugal | [ark:/87602/m4/606348](http://n2t.net/ark:/87602/m4/606348) | http://n2t.net/ark:/87602/m4/606348 |
| NDGS 10019 | Right Maxilla | [ark:/87602/m4/606352](http://n2t.net/ark:/87602/m4/606352) | http://n2t.net/ark:/87602/m4/606352 |
| NDGS 10019 | Right Opisthotic | [ark:/87602/m4/606353](http://n2t.net/ark:/87602/m4/606353) | http://n2t.net/ark:/87602/m4/606353 |
| NDGS 10019 | Right Palatine | [ark:/87602/m4/606357](http://n2t.net/ark:/87602/m4/606357) | http://n2t.net/ark:/87602/m4/606357 |
| NDGS 10019 | Right Parietal | [ark:/87602/m4/606360](http://n2t.net/ark:/87602/m4/606360) | http://n2t.net/ark:/87602/m4/606360 |
| NDGS 10019 | Right Postorbital | [ark:/87602/m4/606363](http://n2t.net/ark:/87602/m4/606363) | http://n2t.net/ark:/87602/m4/606363 |
| NDGS 10019 | Right Prefrontal | [ark:/87602/m4/606366](http://n2t.net/ark:/87602/m4/606366) | http://n2t.net/ark:/87602/m4/606366 |
| NDGS 10019 | Right Prootic | [ark:/87602/m4/606369](http://n2t.net/ark:/87602/m4/606369) | http://n2t.net/ark:/87602/m4/606369 |
| NDGS 10019 | Right Pterygoid | [ark:/87602/m4/606373](http://n2t.net/ark:/87602/m4/606373) | http://n2t.net/ark:/87602/m4/606373 |
| NDGS 10019 | Right Quadrate | [ark:/87602/m4/606376](http://n2t.net/ark:/87602/m4/606376) | http://n2t.net/ark:/87602/m4/606376 |
| NDGS 10019 | Right Quadratojugal | [ark:/87602/m4/606377](http://n2t.net/ark:/87602/m4/606377) | http://n2t.net/ark:/87602/m4/606377 |
| NDGS 10019 | Right Squamosal | [ark:/87602/m4/606381](http://n2t.net/ark:/87602/m4/606381) | http://n2t.net/ark:/87602/m4/606381 |
| NDGS 10019 | Supraoccipital | [ark:/87602/m4/606384](http://n2t.net/ark:/87602/m4/606384) | http://n2t.net/ark:/87602/m4/606384 |
| NDGS 10019 | Vomer | [ark:/87602/m4/606387](http://n2t.net/ark:/87602/m4/606387) | http://n2t.net/ark:/87602/m4/606387 |
| NDGS 10029 | Basioccipital | [ark:/87602/m4/595976](http://n2t.net/ark:/87602/m4/595976) | http://n2t.net/ark:/87602/m4/595976 |
| NDGS 10029 | Cranium | [ark:/87602/m4/596032](http://n2t.net/ark:/87602/m4/596032) | http://n2t.net/ark:/87602/m4/596032 |
| NDGS 10029 | Left Exoccipital | [ark:/87602/m4/596035](http://n2t.net/ark:/87602/m4/596035) | http://n2t.net/ark:/87602/m4/596035 |
| NDGS 10029 | Left Frontal | [ark:/87602/m4/595979](http://n2t.net/ark:/87602/m4/595979) | http://n2t.net/ark:/87602/m4/595979 |
| NDGS 10029 | Left Jugal | [ark:/87602/m4/595982](http://n2t.net/ark:/87602/m4/595982) | http://n2t.net/ark:/87602/m4/595982 |
| NDGS 10029 | Left Opisthotic | [ark:/87602/m4/596038](http://n2t.net/ark:/87602/m4/596038) | http://n2t.net/ark:/87602/m4/596038 |
| NDGS 10029 | Left Palatine | [ark:/87602/m4/596041](http://n2t.net/ark:/87602/m4/596041) | http://n2t.net/ark:/87602/m4/596041 |
| NDGS 10029 | Left Parietal | [ark:/87602/m4/596047](http://n2t.net/ark:/87602/m4/596047) | http://n2t.net/ark:/87602/m4/596047 |
| NDGS 10029 | Left Postorbital | [ark:/87602/m4/596050](http://n2t.net/ark:/87602/m4/596050) | http://n2t.net/ark:/87602/m4/596050 |
| NDGS 10029 | Left Prootic | [ark:/87602/m4/596053](http://n2t.net/ark:/87602/m4/596053) | http://n2t.net/ark:/87602/m4/596053 |
| NDGS 10029 | Left Pterygoid | [ark:/87602/m4/596056](http://n2t.net/ark:/87602/m4/596056) | http://n2t.net/ark:/87602/m4/596056 |
| NDGS 10029 | Left Quadrate | [ark:/87602/m4/596059](http://n2t.net/ark:/87602/m4/596059) | http://n2t.net/ark:/87602/m4/596059 |
| NDGS 10029 | Left Quadratojugal | [ark:/87602/m4/596067](http://n2t.net/ark:/87602/m4/596067) | http://n2t.net/ark:/87602/m4/596067 |
| NDGS 10029 | Left Squamosal | [ark:/87602/m4/596073](http://n2t.net/ark:/87602/m4/596073) | http://n2t.net/ark:/87602/m4/596073 |
| NDGS 10029 | Parabasisphenoid | [ark:/87602/m4/596071](http://n2t.net/ark:/87602/m4/596071) | http://n2t.net/ark:/87602/m4/596071 |
| NDGS 10029 | Right Exoccipital | [ark:/87602/m4/596104](http://n2t.net/ark:/87602/m4/596104) | http://n2t.net/ark:/87602/m4/596104 |
| NDGS 10029 | Right Opisthotic | [ark:/87602/m4/596111](http://n2t.net/ark:/87602/m4/596111) | http://n2t.net/ark:/87602/m4/596111 |
| NDGS 10029 | Right Parietal | [ark:/87602/m4/596114](http://n2t.net/ark:/87602/m4/596114) | http://n2t.net/ark:/87602/m4/596114 |
| NDGS 10029 | Right Prootic | [ark:/87602/m4/596117](http://n2t.net/ark:/87602/m4/596117) | http://n2t.net/ark:/87602/m4/596117 |
| NDGS 10029 | Right Pterygoid | [ark:/87602/m4/596121](http://n2t.net/ark:/87602/m4/596121) | http://n2t.net/ark:/87602/m4/596121 |
| NDGS 10029 | Right Quadrate | [ark:/87602/m4/596122](http://n2t.net/ark:/87602/m4/596122) | http://n2t.net/ark:/87602/m4/596122 |
| NDGS 10029 | Right Squamosal | [ark:/87602/m4/596129](http://n2t.net/ark:/87602/m4/596129) | http://n2t.net/ark:/87602/m4/596129 |
| NDGS 10029 | Supraoccipital | [ark:/87602/m4/596132](http://n2t.net/ark:/87602/m4/596132) | http://n2t.net/ark:/87602/m4/596132 |
| NDGS 10034 | Left Angular | [ark:/87602/m4/597130](http://n2t.net/ark:/87602/m4/597130) | http://n2t.net/ark:/87602/m4/597130 |
| NDGS 10034 | Left Articular | [ark:/87602/m4/597133](http://n2t.net/ark:/87602/m4/597133) | http://n2t.net/ark:/87602/m4/597133 |
| NDGS 10034 | Left Coronoid | [ark:/87602/m4/597136](http://n2t.net/ark:/87602/m4/597136) | http://n2t.net/ark:/87602/m4/597136 |
| NDGS 10034 | Dentary | [ark:/87602/m4/597139](http://n2t.net/ark:/87602/m4/597139) | http://n2t.net/ark:/87602/m4/597139 |
| NDGS 10034 | Mandible | [ark:/87602/m4/597126](http://n2t.net/ark:/87602/m4/597126) | http://n2t.net/ark:/87602/m4/597126 |
| NDGS 10034 | Left Prearticular | [ark:/87602/m4/597142](http://n2t.net/ark:/87602/m4/597142) | http://n2t.net/ark:/87602/m4/597142 |
| NDGS 10034 | Left Surangular | [ark:/87602/m4/597145](http://n2t.net/ark:/87602/m4/597145) | http://n2t.net/ark:/87602/m4/597145 |
| NDGS 10071 | Plastron | [ark:/87602/m4/597340](http://n2t.net/ark:/87602/m4/597340) | http://n2t.net/ark:/87602/m4/597340 |
| NDGS 10084 | Left Angular | [ark:/87602/m4/597164](http://n2t.net/ark:/87602/m4/597164) | http://n2t.net/ark:/87602/m4/597164 |
| NDGS 10084 | Left Coronoid | [ark:/87602/m4/597167](http://n2t.net/ark:/87602/m4/597167) | http://n2t.net/ark:/87602/m4/597167 |
| NDGS 10084 | Dentary | [ark:/87602/m4/597170](http://n2t.net/ark:/87602/m4/597170) | http://n2t.net/ark:/87602/m4/597170 |
| NDGS 10084 | Mandible | [ark:/87602/m4/597161](http://n2t.net/ark:/87602/m4/597161) | http://n2t.net/ark:/87602/m4/597161 |
| NDGS 10084 | Left Surangular | [ark:/87602/m4/597173](http://n2t.net/ark:/87602/m4/597173) | http://n2t.net/ark:/87602/m4/597173 |
| NDGS 10092 | Pelvic girdle | [ark:/87602/m4/597325](http://n2t.net/ark:/87602/m4/597325) | http://n2t.net/ark:/87602/m4/597325 |
| NDGS 10157 | Right Fibula | [ark:/87602/m4/597261](http://n2t.net/ark:/87602/m4/597261) | http://n2t.net/ark:/87602/m4/597261 |
| NDGS 10329 | Dentary | [ark:/87602/m4/598671](http://n2t.net/ark:/87602/m4/598671) | http://n2t.net/ark:/87602/m4/598671 |
| NDGS 10338 | Left Tibia | [ark:/87602/m4/597267](http://n2t.net/ark:/87602/m4/597267) | http://n2t.net/ark:/87602/m4/597267 |
| NDGS 10361 | Left Femur | [ark:/87602/m4/597192](http://n2t.net/ark:/87602/m4/597192) | http://n2t.net/ark:/87602/m4/597192 |
| NDGS 10435 | Vertebra | [ark:/87602/m4/597272](http://n2t.net/ark:/87602/m4/597272) | http://n2t.net/ark:/87602/m4/597272 |
| NDGS 10441 | Vertebra | [ark:/87602/m4/597277](http://n2t.net/ark:/87602/m4/597277) | http://n2t.net/ark:/87602/m4/597277 |
| NDGS 10511 | Left Tibia | [ark:/87602/m4/597282](http://n2t.net/ark:/87602/m4/597282) | http://n2t.net/ark:/87602/m4/597282 |
| NDGS 10559 | Left Femur | [ark:/87602/m4/597287](http://n2t.net/ark:/87602/m4/597287) | http://n2t.net/ark:/87602/m4/597287 |
| NDGS 10560 | Right Tibia | [ark:/87602/m4/597292](http://n2t.net/ark:/87602/m4/597292) | http://n2t.net/ark:/87602/m4/597292 |
| NDGS 10613 | Left Fibula | [ark:/87602/m4/597297](http://n2t.net/ark:/87602/m4/597297) | http://n2t.net/ark:/87602/m4/597297 |
| NDGS 10625 | Carapace | [ark:/87602/m4/597355](http://n2t.net/ark:/87602/m4/597355) | http://n2t.net/ark:/87602/m4/597355 |
| NDGS 11481 | Claw | [ark:/87602/m4/597302](http://n2t.net/ark:/87602/m4/597302) | http://n2t.net/ark:/87602/m4/597302 |
| NDGS 11531 | Right Humerus | [ark:/87602/m4/597310](http://n2t.net/ark:/87602/m4/597310) | http://n2t.net/ark:/87602/m4/597310 |
| NDGS 11788 | Left Coronoid | [ark:/87602/m4/597184](http://n2t.net/ark:/87602/m4/597184) | http://n2t.net/ark:/87602/m4/597184 |
| NDGS 11788 | Dentary | [ark:/87602/m4/597187](http://n2t.net/ark:/87602/m4/597187) | http://n2t.net/ark:/87602/m4/597187 |
| NDGS 11788 | Mandible | [ark:/87602/m4/597181](http://n2t.net/ark:/87602/m4/597181) | http://n2t.net/ark:/87602/m4/597181 |
| NDGS 11879 | Carapace | [ark:/87602/m4/597360](http://n2t.net/ark:/87602/m4/597360) | http://n2t.net/ark:/87602/m4/597360 |
| NDGS 1203 | Carapace | [ark:/87602/m4/597335](http://n2t.net/ark:/87602/m4/597335) | http://n2t.net/ark:/87602/m4/597335 |
| NDGS 18191 | Pectoral girdle | [ark:/87602/m4/597345](http://n2t.net/ark:/87602/m4/597345) | http://n2t.net/ark:/87602/m4/597345 |
| NDGS 18192 | Left Radius | [ark:/87602/m4/597315](http://n2t.net/ark:/87602/m4/597315) | http://n2t.net/ark:/87602/m4/597315 |
| NDGS 19193 | Vertebra | [ark:/87602/m4/597320](http://n2t.net/ark:/87602/m4/597320) | http://n2t.net/ark:/87602/m4/597320 |

| µCT Slices | | | |
| --- | --- | --- | --- |
| Catalog Number | Description |  | ARK Link |
| NDGS 10019 | Cranium | [ark:/87602/m4/594391](http://n2t.net/ark:/87602/m4/594391) | http://n2t.net/ark:/87602/m4/594391 |
| NDGS 10029 | Cranium | [ark:/87602/m4/595972](http://n2t.net/ark:/87602/m4/595972) | http://n2t.net/ark:/87602/m4/595972 |
| NDGS 10034 | Mandible | [ark:/87602/m4/597123](http://n2t.net/ark:/87602/m4/597123) | http://n2t.net/ark:/87602/m4/597123 |
| NDGS 10084 | Mandible | [ark:/87602/m4/597158](http://n2t.net/ark:/87602/m4/597158) | http://n2t.net/ark:/87602/m4/597158 |
| NDGS 11788 | Mandible | [ark:/87602/m4/597178](http://n2t.net/ark:/87602/m4/597178) | http://n2t.net/ark:/87602/m4/597178 |
